# Neurobiologically informed graph theory analysis of the language system

**DOI:** 10.1162/netn_a_00443

**Published:** 2025-04-30

**Authors:** Yosuke Morishima, Martijn van den Heuvel, Werner Strik, Thomas Dierks

**Affiliations:** Translational Research Center, University Hospital of Psychiatry and Psychotherapy, University of Bern, Bern, Switzerland; Department of Complex Trait Genetics, Center for Neurogenomics and Cognitive Research, Amsterdam Neuroscience, Vrije Universiteit Amsterdam, Amsterdam, The Netherlands; Department of Child and Adolescent Psychiatry and Psychology, Section Complex Trait Genetics, Amsterdam Neuroscience, Vrije Universiteit Medical Center, Amsterdam University Medical Center, Amsterdam, The Netherlands

**Keywords:** Network hierarchy, fMRI, Language, Task dependency, Adaptive behavior

## Abstract

Recent advancements in neuroimaging data analysis facilitate the characterization of adaptive changes in brain network integration. This study introduces a distinctive approach that merges knowledge-informed and data-driven methodologies, offering a nuanced way to more effectively understand these changes. Utilizing graph network analysis, along with existing neurobiological knowledge of domain-specific brain network systems, we uncover a deeper understanding of brain network interaction and integration. As a proof of concept, we applied our approach to the language domain, a well-known large-scale network system as a representative model system, using functional imaging datasets with specific language tasks for validation of our proposed approach. Our results revealed a double dissociation between motor and sensory language modules during word generation and comprehension tasks. Furthermore, by introducing a hierarchical nature of brain networks and introducing local and global metrics, we demonstrated that hierarchical levels of networks exhibit distinct ways of integration of language brain networks. This innovative approach facilitates a differentiated and thorough interpretation of brain network function in local and global manners, marking a significant advancement in our ability to investigate adaptive changes in brain network integration in health and disease.

## Author Summary

This study introduces a novel approach combining knowledge-informed and data-driven methodologies to analyze adaptive changes in brain network integration. By integrating graph network analysis with existing neurobiological knowledge of domain-specific brain networks, we provide deeper insights into brain network interaction, coordination, and integration. Using the language domain as a model system and functional imaging data from specific language tasks, our approach reveals a double dissociation between motor and sensory language modules during word generation and comprehension tasks. By analyzing hierarchical networks at multiple levels and introducing local and global metrics, we demonstrate that hierarchical levels of networks exhibit distinct integration patterns. This method surpasses conventional regional activation analysis, offering a significant advancement in investigating adaptive changes in brain network integration in health and disease.

## INTRODUCTION

Historically, studies on brain functions investigated local brain lesions due to stroke or surgery to correlate brain areas with cognitive functions (Broca, 1861; Finger, 2001; Penfield & Boldrey, 1937). Early brain imaging studies also focused on circumscribed brain areas (Poldrack, 2010; Price & Friston, 2005). However, brain systems are not defined by one single brain area but by the coordination of distributed brain areas to process information in favor of a behavioral goal at hand (Barbey, 2018; Desimone & Duncan, 1995; Miller & Cohen, 2001). Therefore, recent studies have emphasized network aspects of the brain systems and their association with cognitive processes, behavior, and illness (Bullmore & Sporns, 2012; Fornito et al., 2015; He & Evans, 2010; Park & Friston, 2013; Rubinov & Sporns, 2010; Stephan et al., 2009).

In brain research, structural and functional neuroimaging methods have been used to assess brain networks in vivo. Two more or less distinct approaches are utilized to investigate brain network functions. The first class of brain network analysis exploits a priori knowledge derived from extensive studies on functional localizations in brain systems. In other words, we call it a knowledge-informed (in general terms, a top-down) approach. This approach investigates how particular brain areas are connected with other brain areas (e.g., seed-based connectivity).

More recently, the second class of brain network analysis investigates brain imaging data without constraints on a particular brain circuit but rather defines networks derived from these data (e.g., independent component analysis and clustering). They can be referred to as data-driven (or in general terms, a bottom-up) approach. Recent applications of graph network analysis to brain systems have opened opportunities to understand network features (Bullmore & Sporns, 2012; Fornito et al., 2015; He & Evans, 2010; Rubinov & Sporns, 2010). Graph network analysis has mainly been applied to the data-driven approach to characterize whole-brain networks in healthy individuals, development, aging, neurological, and psychiatric disorders (Bassett et al., 2008; Brier et al., 2014; Chiang & Haneef, 2014; Finotelli et al., 2018; Khazaee et al., 2015; Rocca et al., 2016; Supekar et al., 2009; van den Heuvel et al., 2013; van den Heuvel & Sporns, 2013). However, the approach can be applied to bridge knowledge-informed and data-driven approaches of network analysis. Such an approach to integrate knowledge-informed and data-driven network analysis has largely been missing to provide a more differentiated and comprehensive understanding of brain network interaction and integration.

We here aim to fill the gap between the knowledge-informed and data-driven approaches. We exploit existing neurobiological knowledge of domain-specific brain network systems (Catani & De Schotten, 2012; K. L. Campbell & Tyler, 2018; Kim et al., 2012; Mahon, 2022; Morey et al., 2019; Yeo et al., 2011) and apply graph network analysis to how the domain-specific brain network systems are integrated. As the brain networks are organized in a hierarchical way (Felleman & Van Essen, 1991; Park & Friston, 2013), we will investigate multiple hierarchical levels of domain-specific brain networks.

We propose an approach investigating domain-specific brain network systems by using graph network analysis. To test our approach, we focused on the language domain, a well-described large-scale network systems (Ardila et al., 2016; Catani & De Schotten, 2012; Friederici, 2011; Rauschecker & Scott, 2009; Salmelin & Kujala, 2006; Skeide & Friederici, 2016), and explored language network integration with a publicly available and validated functional imaging dataset with specific language task.

We propose that these cognitive, here, language, tasks can be mapped upon the corresponding specific parts of the language system using graph theory analysis.

This approach can reveal functional properties of the neural circuits that were not previously available, especially when compared with conventional fMRI analysis, which primarily measures the amount of activity in circumscribed regions.

Historically, functional parcellation of brain areas has been performed based on the microscopic characteristics of cortical layers (Brodmann, 1909; A. W. Campbell, 1905). Although Brodmann’s parcellation of brain areas (Brodmann area, BA) is not fully supported by more recent research, it is still widely used to register functional activation to anatomical structures (Amunts et al., 2014; Zilles & Amunts, 2010). In the current study, we use BA as a base unit of functional modules and add some modifications to take into account more recent research. As brain networks are hierarchically integrated (Felleman & Van Essen, 1991; Park & Friston, 2013), we specify functional modules at several hierarchical levels, from a local functionally distinct level to a global functionally integrated level.

As a proof-of-concept study, the current study aims to validate our approach integrating knowledge-informed and data-driven network analysis, and we performed two independent analyses using publicly available fMRI datasets from OpenNeuro (openneuro.org). While language is used as an example in this analysis, the method can be broadly applied to various brain circuits beyond language processing. The first analysis examined how functionally specialized brain network systems were integrated when executing various cognitive tasks requiring the networks, focusing on language brain networks (Ardila et al., 2016; Skeide & Friederici, 2016), utilizing two language tasks: word comprehension (WC) and word generation (WG) tasks. We hypothesize that graph metrics would exhibit interaction effects between the two language tasks and the motor and sensory network modules. The second analysis, as validation, examined how the somatosensory-motor (SM) cortex was differently integrated between the right and left hemisphere, using a simple motor task fMRI dataset.

## METHODS

### Sources of Task fMRI Datasets and Cognitive Tasks

We used two publicly available open MRI datasets in the current study. We used OpenNeuro dataset ds004073 “Comparing language lateralization using fMRI and fTCD” (https://doi.org/10.18112/openneuro.ds004073.v1.0.0) (COLA Consortium et al., 2022). The dataset includes six language tasks, and we used the WC and the WG tasks. In the WC task, participants were visually presented with two pictures and indicated which picture matched an acoustically presented word. In the WG task, participants performed a phonetic verbal fluency task, requiring them to silently generate words starting with a letter indicated (e.g., “s,” “l,” etc.). The Oxford language dataset includes 50 subjects, but here, one subject was excluded due to missing WC task data, and two were excluded due to the corruption of fieldmap images.

For a validation analysis, another dataset from OpenNeuro was used, the “A test-retest fMRI dataset for motor, language and spatial attention functions” (https://openneuro.org/datasets/ds000114/versions/1.0.2) dataset, of which the motor task data were used. During the simple motor task, participants were required to move either their right index finger, right foot, or lips for 15 s according to the instructions. Interblock intervals were set to 15 s. The dataset included 10 subjects. We used both “test” and “re-test” data to examine the consistency of results.

### Preprocessing of MRI Data and Computing Connectivity Matrix

We first preprocessed MRI data with SPM12 (https://www.fil.ion.ucl.ac.uk/spm/). Structural images were processed by the CAT12 toolbox (https://neuro-jena.github.io/cat/), and a deformation field to normalize MRIs from an individual native space to the MNI template space was determined. Functional images were corrected for distortion due to magnetic inhomogeneity using field map b0 images and realigned to correct head movements. Then, the functional images were coregistered to a structural image in a native individual space. Next, the coregistered functional images were normalized into the MNI space, and then spatial smoothing was applied with a Gaussian filter of 4 mm full-width half maximum.

To extract time series from fMRI datasets, we defined 1,054 nodes, which consist of 1,000 cortical nodes and 54 subcortical nodes. Cortical nodes were defined by using Schaefer’s cortical parcellation atlas (Schaefer et al., 2018). We used the MNI-registered 1,000-parcel mask image. For subcortical nodes, we used the finest resolution (Level 4 resolution including 54 parcels) of the Melbourne subcortical atlas (Tian et al., 2020). We then extracted time series from each node. Six head movement parameters, cerebrospinal fluid (CSF) and white matter (WM) signals, and their first-order derivatives were used to remove artifacts. In addition, we scrubbed the volumes with excessive head movement by using a 0.5-mm threshold of framewise displacement (Power et al., 2014) and one back and two forward neighbors. Mean percentages of excluded volumes were 6.3% and 10.6% for the WC and WG tasks, respectively. There was a significantly higher percentage of volumes that was excluded in the WG task (*p* = 0.007). We applied filtering with six head movement parameters and their first-order derivatives and lastly applied high-pass filtering of 0.008 Hz.

The connectivity matrix was calculated by Pearson’s correlation of the preprocessed time series. For each connectivity matrix, a threshold of top 20%, 30%, or 40% was applied to compute the binary connectivity matrix. When global signal scaling was included, we calculated the partial correlation of preprocessed time series controlled by global signal, the average of all voxels including gray matter, WM, and CSF.

### Language Functional Module

The architecture of language network modules was defined based upon literature (Ardila et al., 2016; Catani & De Schotten, 2012; Friederici, 2011; Rauschecker & Scott, 2009; Salmelin & Kujala, 2006; Skeide & Friederici, 2016) and comprised four levels of spatial resolution and hierarchy (see Table 1). Thus, the following BAs were included in the language system: BA20, BA21, BA22, BA38, BA39, BA40, BA41, BA42, BA44, BA45, and BA47. BA21 and BA22 were further divided into anterior and posterior parts by the median *y* coordinate of these areas. Next, we calculated the center coordinate of each node and assigned each area or outside of the language module. Here, note that BAs are not the minimal units of network nodes. Instead, each of the 1,054 nodes, comprising 1,000 cortical and 54 subcortical nodes, represents the minimal unit of a network node. Ten functional modules are included in the finest resolution of the language module architecture (Level 1 modules). Each module contains several Broadman areas separately organized by left and right hemispheres. Based on the literature, the “Word recognition” module includes the BA20 and the posterior part of BA21 and BA22. The “Semantic processing” module includes BA38 and the anterior part of BA21 and BA22. The “Syntactic processing” module includes BA39 and BA40. The “Auditory processing” module includes BA41 and BA42. The last “Motor language” module includes BA44, BA45, and BA47. The second-level module includes six modules. The “Word processing” module consists of “Word recognition” and “Semantic processing” modules. The “Language sensory” module consists of “Semantic processing” and “Auditory processing” modules. The “Language motor” module is identical to the “Motor language” module. The third level consists of two modules: all areas in the language module, either in the right or left hemisphere. The last fourth level corresponds to the entire network in the language module.

**Table 1.** Language module specification

Level 1	Level 2	Level 3	Level 4
L/R separated			L/R combined
Word recognition (BA20, Post BA21, Post BA22)	Word processing	Language network	Language network
Semantic processing (Ant BA21, Ant BA22, BA38)			
Syntactic processing (BA39, BA40)	Language sensory		
Auditory processing (BA41, BA42)			
Motoric Language (BA44, BA45, BA47)	Language motor		

### Graph Metrics

We calculated two different ways to compute graph metrics of functional modules. First, we computed “local metrics,” which represent how brain nodes are integrated within a functional module. Here specifically, graph metrics were calculated using all nodes in each functional module and then averaged across the nodes. Second, we computed “global metrics,” which represent how brain nodes of each functional module are integrated into a whole brain network by considering connections to nodes outside of the functional module. Graph metrics were calculated using the whole brain network (1,054 nodes) and averaged across all nodes within each functional module. We calculated the graph metrics using the Brain Connectivity Toolbox (https://sites.google.com/site/bctnet/) (Rubinov & Sporns, 2010). Graph metrics calculated are listed in Table 2. We used metrics derived from a binary graph as the main results. The graph metrics we calculated were exhaustive but redundant. Among the metrics we calculated, quite a few metrics were correlated with each other (Supporting Information Figure S1). Therefore, we present the following three metrics as our main results: degree density, average shortest path length, and betweenness centrality (Oehlers & Fabian, 2021). Degree density refers to the average number of connections within a network scaled by the total number of nodes in the network. A higher degree density generally indicates more connections between nodes, making the network not only more robust against random failures but also more vulnerable to targeted attacks. We use this metric as a measure of network connectivity and stability. The shortest path length refers to the minimum distance between a pair of nodes. Shorter path lengths directly relate to shorter transmission times. We use the average shortest path length as a measure of the network’s efficiency in transmitting information. Betweenness centrality calculates the fraction of shortest paths between all node pairs that pass through a given node or edge. A node or edge with higher betweenness centrality is likely to carry more traffic in shortest path routing scenarios. We thus use betweenness centrality as a measure of traffic load. The comprehensive results of graph metrics and statistical comparison between the WC and WG tasks are listed in Supporting Information Tables S3 and S5. As an additional control analysis, we included global functional connectivity (FC) value (mean of whole brain FC) for each task and subject to control the overall FC (Supporting Information Tables S4 and S6).

**Table 2.** List of graph metrics

	Global	Local
Degree density	+	+
Shortest path length	+	+
Clustering coefficient	+	+
Nodal efficiency	+	+
Betweenness centrality	+	+
Eigenvalue centrality	+	+
Assortativity		+
Participation coefficient	+	

### Further Validation Analysis

As a validation, we conducted three validation analyses: comparison against resting-state fMRI data, whole-brain node randomization, and simple motor task fMRI data. For resting-state comparison, we used preprocessed resting-state fMRI data from 100 unrelated subjects in the Human Connectome Project (https://www.humanconnectome.org/). Following the same procedure used for the language task fMRI data, we extracted a time series of 1,054 nodes and calculated correlation matrices. To assess the robustness of our results, we performed bootstrap resampling of connectivity matrices followed by application of 30% of relative threshold to create a binary graph. Then, we calculated graph metrics of the word recognition, motoric language, and left language modules. We repeated this procedure for 10,000 times. From these bootstrap distributions, we derived *p* values for each language task condition. This procedure allowed us to quantitatively estimate the variability and statistical significance of observed network differences. Second, we performed whole-brain node randomization analysis to validate the specificity of task-dependent changes in the network architecture of the language network modules. To this end, we randomly selected nodes to create a pseudofunctional module. For each WC or WG task, we created a template connectivity matrix by averaging an individual connectivity matrix across subjects. Then, 30% of relative threshold was applied to create a binary graph of each task. To match the language module we have used, we randomly selected the same number of nodes matched to the word recognition, motoric language, and left language modules. Then, local and global density, shortest path length, and betweenness centrality were calculated for each of WC or WG task. We repeated this procedure for 10,000 times to obtain distributions of graph metrics derived from random null functional modules. As further validation, another dataset of a simple motor task was used. The simple motor task involved right finger, foot, or lips movements in a blocked design. We here specified an SM module, including BAs 1, 2, 3, and 4. We compared the graph metrics of the left and right SM modules.

### Statistical Analysis

To compare the graph metrics between tasks or between halves of time series, we performed a paired *t* test. To study the interaction effect between tasks and modules, we performed a repeated-measures two-way analysis of variance for conventional activation and linear mixed-effects model for graph metrics. In linear mixed-effects model analysis, we included functional module, language task, and their interaction effects in a fixed global correlation (when included) as a fixed-effect factor, and a by-subject factor random effect. To account for the difference in global correlations, we also included a global correlation for each task and each subject as a fixed-effect factor. Correction for multiple comparison was performed with false discovery rate (FDR) correction.

## RESULTS

To demonstrate the functional specificity of language-related functional modules regarding BOLD activation, we initially investigated task-related activation. First, we focused on the “Word recognition” and “Motoric language” modules at Level 1 during the WC and WG tasks (Figure 2A and Table 1). In the “Word recognition” module, activation during the WC task was expected to be higher during the WG task, while vice versa in the “Motoric language” module. Therefore, Task × Functional module interactions were expected. A significant interaction between Task × Functional module interactions (*F*(1, 45) = 37.76, *p* < 0.0001) (Figure 2B) was found. In the “Word recognition” module, higher activation during the WC task compared with the WG task was observed. Conversely, in the “Motoric language” module, higher activation was observed during the WG task compared with the WC task.

**Figure 1.**
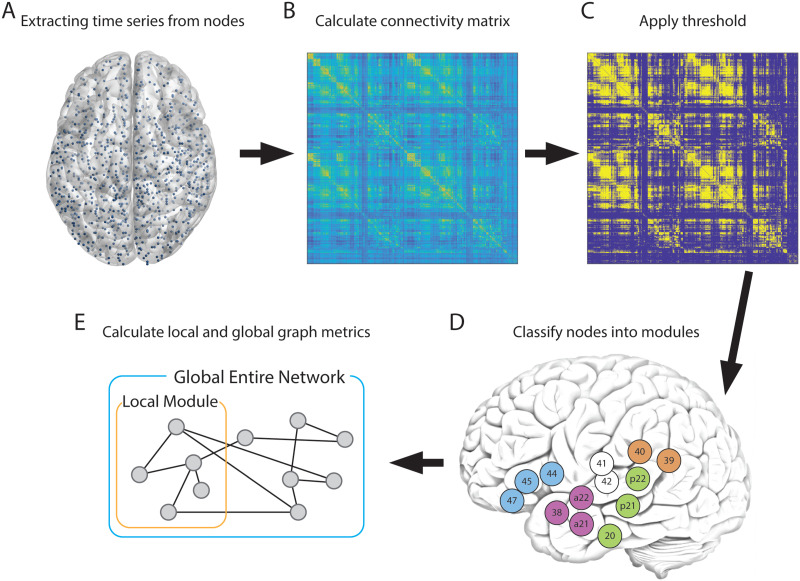
Data processing pipeline. (A) All 1,054 nodes across the whole brain except the cerebellum are defined. The center coordinate of each node is depicted by a blue dot. For each node, BOLD time series were extracted, and cleaned nuisance covariates and cleaned noisy volumes. (B) A connectivity matrix is computed from the extracted BOLD time series. (C) The connectivity matrix is binarized by a relative threshold. (D) nodes are classified to functional modules (Table 1, colors represent functional modules of the language system and numbers BA). (E) Local and global graph metrics are calculated based on functional module specifications. Local graph metrics are calculated only with nodes in a particular functional module. Global graph metrics are calculated with all 1,054 nodes in the whole brain.

**Figure 2.**
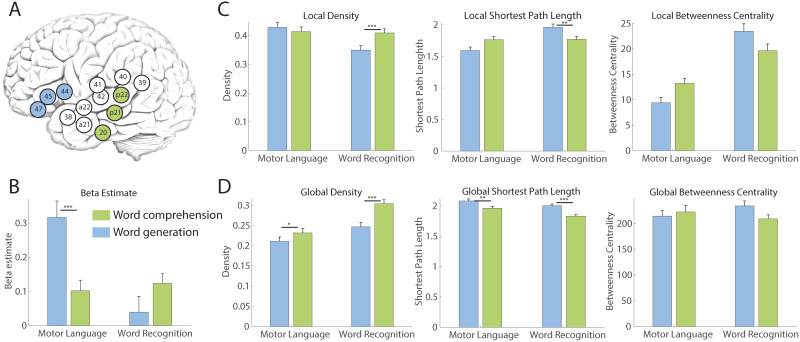
BOLD activation and graph metrics in word recognition and motoric language modules at Level 1 during the language tasks. (A) Definition of word recognition (green) and motoric language (blue) functional modules. (B) Averaged beta estimate of all voxels in the functional modules for the WC (green) and WG (blue) tasks showing significant interaction effects. (C) Local graph metrics showed significant interactions between the task and functional module. (D) Global graph metrics during the WC and WG tasks. These graph metrics were obtained without global scaling. Bars indicate standard deviation. *p < 0.05, **p < 0.01, ***p < 0.001 for post hoc comparison between the WC and WG tasks.

Subsequently, to examine network integrations in those functional modules beyond the simple activation patterns described above, we tested whether consistent interaction effects in graph metrics were observed. According to our analysis pipeline (Figure 1), the graph metrics in the “Word recognition” and “Motoric language” modules during the WC and WG tasks were calculated (Figure 2). Local graph metrics were computed using only nodes within a functional module and ignoring nodes outside of the functional module to characterize the integrity of the network in each functional module (see details in the Methods section). For these local graph metrics, density, average shortest path length, and betweenness centrality demonstrated a significant interaction between language tasks and motor-sensory language modules (density, *t* = 2.834, *p* = 0.005; shortest path length, *t* = −2.192, *p* = 0.002; betweenness centrality, *t* = −1.573, *p* = 0.117) (Figure 2C). As anticipated, the higher density and shortest path length in the “Motoric language” module were observed during the WG task than during the WC task. Concerning betweenness centrality, we discovered lower betweenness centrality in the “Motoric language module” during the WG task compared with the WC task, and the opposite in the “Word recognition” module. This could be explained by the following reason: Due to the shorter path length and higher level of connectedness in high-density networks, betweenness centrality measures tend to be relatively lower.

For the global graph measures, we characterized how a functional brain module additionally communicates with the outside of the functional module (see details in the Methods section) (Figure 2D). Global node degree was a significant main effect of tasks, higher in the WC task than the WG task (*t* = −3.395, *p* = 0.0008), but did not show a significant interaction effect (*t* = 1.644, *p* = 0.102). Shortest path length was shorter in the WC task than the WG task (*t* = 4.937, *p* < 0.0001), but did not show a significant interaction effect (*t* = −0.296, *p* = 0.767). Betweenness centrality did not show the significant main effect of task and interaction effect (Supporting Information Table S1). There is an ongoing debate whether global signals need to be controlled for in functional connectivity analysis (Liu et al., 2017; Murphy & Fox, 2017). Therefore, we calculated the connectivity matrix with and without global signal scaling and compared the interactions between functional modules and tasks. We found that the interactions between modules and tasks were consistent regardless of global scaling. However, the differences between the language tasks were weakened in most of graph metrics (Supporting Information Table S2).

**Figure 3.**
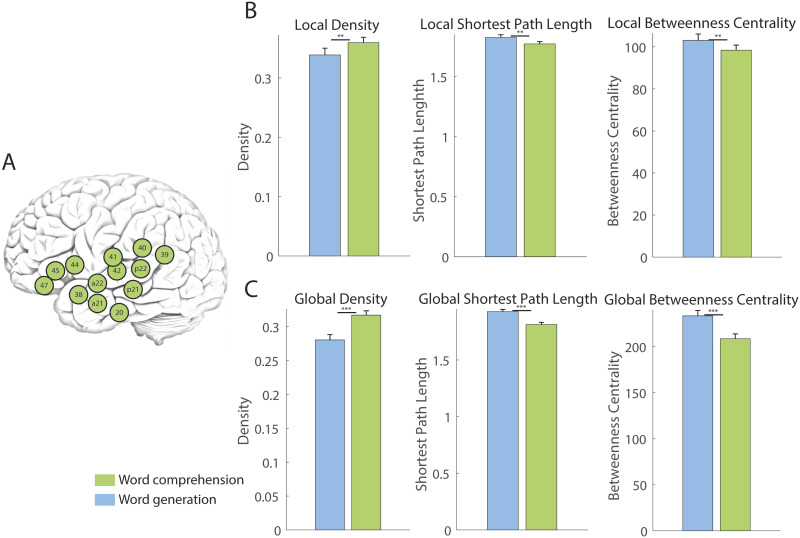
Graph metrics in the left language modules at Level 3 during the language tasks. (A) Definition of left language module. (B) Local graph metrics showed a significant difference between the WC (green) and WG (blue) tasks in the left language module. (C) Global graph metrics during the WC (green) and WG (blue) tasks in the left language module. These graph metrics were obtained without global scaling. Bars indicate standard deviation. *p < 0.05, **p < 0.01, ***p < 0.001 for post hoc comparison between the WC and WG tasks.

Next, we examined how the levels of hierarchical brain systems influence brain network integration. For example, functional modules at Level 1 are more locally constrained and thus exhibit greater functional distinction, whereas those at Level 3 encompass those functionally distinct and long-distant areas within a single functional module. This multiscale hierarchical approach enables us to determine whether the execution of a cognitive task necessitates more locally constrained or distributed brain areas. We assessed the left language module at Level 3, which included all language modules in the left hemisphere (Figure 3A and Table 1). For local graph metrics, density was significantly higher in the WC task than in the WG task (*t*(45) = −3.058, *p* = 0.003), while shortest path length and betweenness centrality were lower in the WC task than the WG task (shortest path length, *t*(45) = 3.372, *p* = 0.0015; betweenness centrality, *t*(45) = 3.072, *p* = 0.0036) (Figure 3B, Supporting Information Tables S3 and S4). The results indicate that the entire language networks in the left hemisphere are more integrated during the WC task than the WG task. For global graph metrics, density was significantly higher in the WC task (*t*(45) = 5.287, *p* < 0.0001), and shortest path length and betweenness centrality were significantly lower in the WC task (shortest path length, *t*(45) = 5.182, *p* < 0.0001; betweenness centrality, *t*(45) = 4.614, *p* < 0.0001). The results indicate that all language networks in the left hemisphere are more integrated with brain areas outside of the left language networks in the WC task. Comprehensive measures of all graph metrics in all functional modules are shown in Supporting Information Tables S3 and S4.

**Figure 4.**
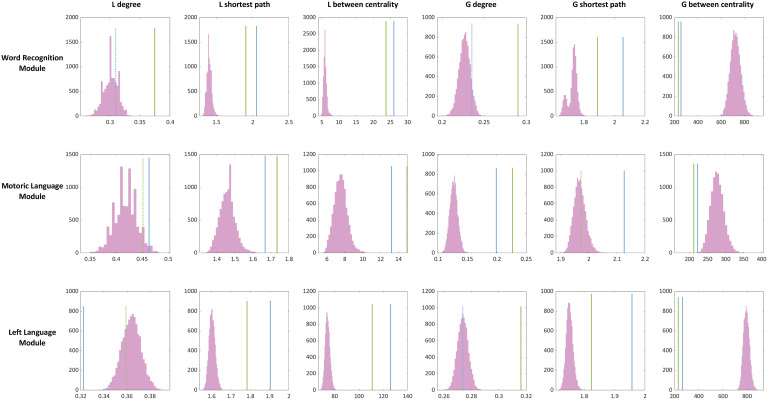
Comparison of language tasks and resting condition. Histogram plots (magenta) represent bootstrap distribution of graph metrics derived from resting-state condition. Line plots represent across subject mean value of graph metrics from the language tasks. Green and blue line plots correspond to the WC and WG task, respectively. Solid line plots indicate that the observed mean value differs significantly from the distribution of the random null functional module (*p* < 0.05, corrected for multiple comparisons using FDR). Dotted line plots indicate that the observed mean value is not significantly different (*p* (FDR) > 0.05).

**Figure 5.**
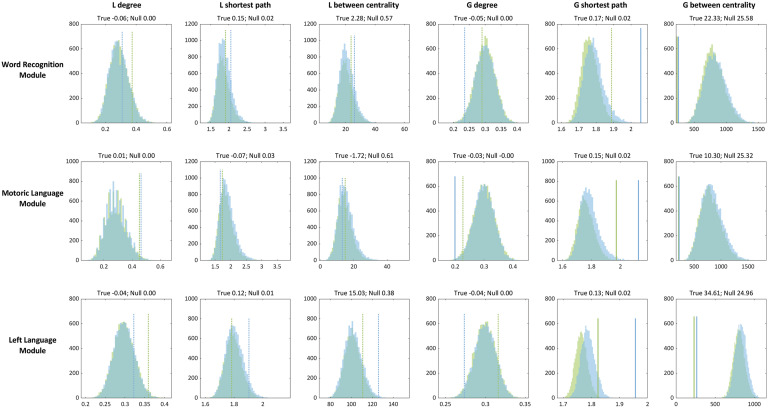
Comparison of the language module and random null module. Histogram plots represent distribution of graph metrics derived from random null functional module. Line plots represent across subject mean value of graph metrics from true functional modules. The numbers above each plot correspond to difference of mean value between the two language tasks derived from the true functional module (true) and random null functional module (null), respectively. For both histogram and line plots, green and blue correspond to the WC and WG task, respectively. Solid line plots indicate that the observed mean value differs significantly from the distribution of the random null functional module (*p* < 0.05, corrected for multiple comparisons using FDR). Dotted line plots indicate that the observed mean value is not significantly different (*p* (FDR) > 0.05).

To validate our observations of network integration in the functional language modules, we conducted two analyses. First, we compared the graph metrics from the language tasks with those derived from resting-state fMRI data. We found a distinctive pattern of differences: while local density in most of the language modules did not significantly differ from the resting-state condition, local shortest path lengths, local and betweenness centrality, and most of global graph metrics were all significantly altered during the language tasks (Figure 4; *p* (FDR) < 0.05). These results suggest that, although certain local characteristics remain stable, the functional language modules assume a more integrated network configuration when engaged in active language tasks. Second, we performed node randomization, created random null functional modules, and then calculated graph metrics for each functional module and each language task. Figure 5 visualized the true values of graph metrics derived from the language modules (lines in Figure 5) and the distribution of graph metrics derived from random null functional modules (histograms in Figure 5). First, the mean values of global betweenness centrality and shortest path length across subjects derived from the true language functional modules were significantly different from the distribution of null functional modules (*p* < 0.05, FDR corrected). These results suggest that that the current approach captures task-dependent changes in domain-specific network integration in particular out-bound network interactions.

**Figure 6.**
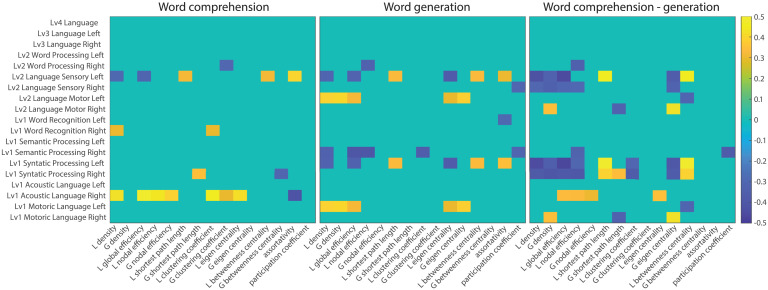
Association between regional BOLD activation and graph metrics. Correlation coefficients between BOLD activation and graph metrics in each functional module are visualized in the WC task (left), WG task (middle), and the difference between WC and WG tasks (right). L and G represent local and global graph metrics, respectively. For visualization purposes, correlation coefficients are thresholded at a *p* value less than 0.05.

In the current study, we observed interaction effects between tasks and functional modules in graph metrics as well as BOLD activation. It is worth further investigating whether task-dependent changes in the regional BOLD activation are associated with task-dependent changes in graph metrics. To this end, we examined if the BOLD activation was correlated with the graph metrics for each task, and if the difference in the BOLD activation between the two tasks was correlated with the difference in the graph metrics between the two tasks. Some significant correlations with a liberal threshold at *p* < 0.05 were observed (Figure 6), but no systematic pattern of significant correlations was observed. Instead, they gave an impression of randomness. In fact, none of the correlations survived at the FDR corrected threshold at *p* < 0.05. Thus, the results suggest that BOLD activation cannot predict graph metrics, and BOLD activation and graph metrics capture distinct aspects of brain network functions.

In the current study, we employed the relative thresholding approach to construct binary networks. However, as noted (van den Heuvel et al., 2017), this approach can result in biased findings. First, choosing a specific threshold could pick random false positive or negative results. Second, global FC, derived from the average correlation across whole brain networks, could create the systemic bias of connections (van den Heuvel et al., 2017). Concerning the first problem, we chose 30% of the relative threshold as representative results, as shown in Figures 2 and 3. We also conducted the same analysis with 20% and 40% of the relative threshold (Supporting Information Table S1). The overall trends of the results were consistent with those obtained at the 30% relative threshold. To address the second problem, we compared global FC values between the WC and WG tasks and found that global FC was significantly higher in the WG than the WC task (WG, 0.330; WC, 0.288; *t*(49) = 4.52, *p* = 0.0004). Therefore, we further performed Task × Module comparisons adjusted by global FC values (Supporting Information Tables S5 and S6). We still observed the consistent pattern of interactions between tasks and modules regardless of adjustment of global FC values in both with or without global scaling.

**Figure 7.**
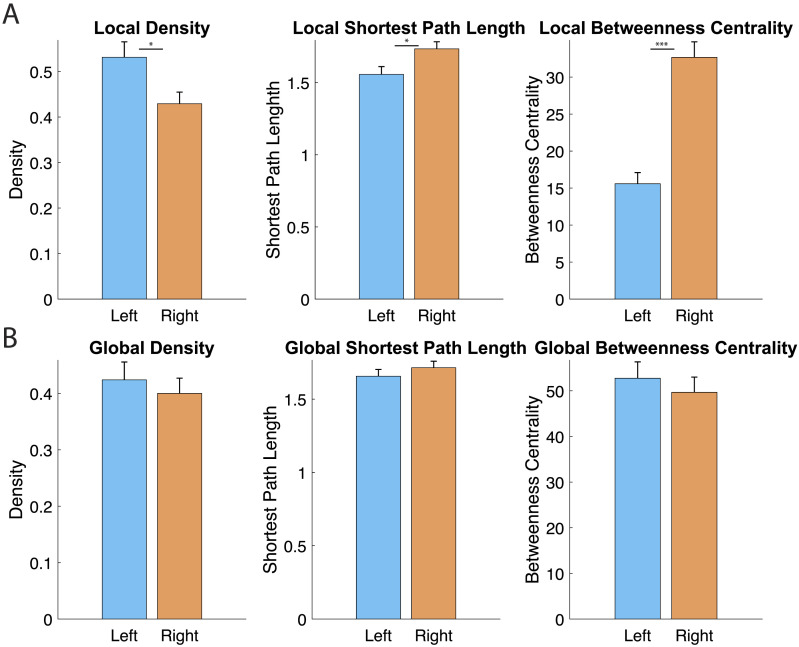
Graph metrics in the somatosensory motor module during the simple motor task (right finger, right foot and lips movements). (A) Local graph metrics showed a significant difference between the left (blue) and right (orange) somatosensory-motor modules (*t*(9) > 2.73; *p* < 0.024). (B) Global graph metrics the left (blue) and right (orange) somatosensory-motor modules were not significantly different (*p* > 0.05). Bars indicate standard deviation.

Lastly, we further validated the current pipeline. To this end, we utilized data from a simple motor task to avoid the complexities of higher order cognitive processes and intricate possibilities of data interpretation. In this task, subjects performed lips, right finger, or foot movement during the fMRI scanning. It was presumed that the left somatosensory and motor areas are more active than the right areas. Consistent with this prediction, we found that significantly higher local density, local shortest path length, and lower betweenness centrality in the left SM module than those of the right SM module (*t*(9) > 2.73; *p* < 0.024; Figure 7, Supporting Information Table S7). In contrast, global graph metrics were not significantly different (*p* > 0.05). These results were replicated with the re-test data (Supporting Information Table S7). These results confirm that local metrics are more consistent with regional activation, while global metrics capture distributed network integrations.

## DISCUSSION

In the present study, we aimed to combine existing knowledge of functional specialization in the brain with graph network analysis to provide a more differentiated and comprehensive understanding of brain network interaction and integration in contrast to conventional fMRI “activity” analysis and data-driven functional connectivity approaches. To demonstrate a proof of concept, we here exploited extensive prior literature on language brain network and specified network modules (Table 1) (Friederici, 2011; Salmelin & Kujala, 2006; Skeide & Friederici, 2016). Using two simple language tasks, WG and WC, we found a double dissociation between the motor and sensory language modules and the graph metrics of the WG and WC tasks, allowing to capture further functional properties of brain network integrations. Additionally, we calculated graph metrics at multiple hierarchical levels of network modules. Lastly, we performed validation analyses to confirm that graph metrics captured network integration in random null functional module as well as in an independent task setting.

The approach has several advantages. First, we predefined specialized functional modules—circuits—using existing knowledge of brain functions rather than defining them based on the clustering of connectivity data. This allows us to interpret how specific functionally defined brain networks operate, as functional modules serve as interpretable units in relation to cognitive processes. In this study, we examined how language brain networks function during the WG and WC tasks.

Most previous studies defined functional modules across the entire brain. One common approach calculates voxel-to-voxel correlations in the entire brain and applies clustering to define functional modules (Brier et al., 2014). Another approach employs gross anatomical boundaries (e.g., sulci between gyri) (Finotelli et al., 2018; Khazaee et al., 2015; Rocca et al., 2016; Supekar et al., 2009; van den Heuvel et al., 2013; van den Heuvel & Sporns, 2013). While these methods enable more explorative analysis and potentially unexpected findings, they often make interpretation difficult. In contrast, our current approach predefines functional modules already mapped to cognitive processing, making results more logical to interpret in relation to well-established structural and functional large-scale cerebral networks. One study by Bertolero et al. (2015) mapped functional modules derived from resting-state fMRI data to author-topic model cognitive functions in a data-driven manner. Another data-driven network approach is utilizing parcellated networks, such as the default mode network. Graph network properties of the DMN were studied at the structural and functional levels (Fan et al., 2021; Pan et al., 2022). One study examined more functionally defined network properties. Viher et al. (2020) examined motor imagery networks with diffusion tensor imaging (DTI) in patients with psychotic spectrum disorders and found graph metrics of motor imagery networks associated with motor skill impairment in these patients. Due to the limitations of DTI streamline connection analysis, the study could only define coarse nodes. As recent research in psychiatric disorders emphasizes addressing the dysfunction of domain-specific networks (Cuthbert & Insel, 2013; Strik et al., 2018), our current approach will contribute unique insights, furthering the comprehension of brain network dysfunctions in the spectrum of neuropsychiatric disorders.

Second, the current pipeline defined multiple hierarchical levels of functional modules/circuits, ranging from more localized functionally distinct modules (e.g., “Auditory processing” module) to more globally integrated modules (e.g., language left). This approach enables us to study the multiple hierarchical levels of brain network integrity in functional domains of information processing. Previous applications of graph theory to the study of brain network hierarchies have largely concentrated on network clustering (Boly et al., 2012; Schaefer et al., 2018; Wang & Li, 2013; Yeo et al., 2011). However, in this study, we pivot our focus toward understanding how clustered brain networks are adaptively employed. We found that the left language module exhibited considerably higher density and shorter shortest path length during the WC task compared with the WG task. The WC task, which entails matching spoken words with visually presented pictures, demands auditory and visual processing, multimodal integration, access to semantic knowledge, and motor actions. Thus, it requires the integration of widely distributed brain areas. In contrast, the WG task, which involves generating words starting with a particular letter, requires the motoric part of the language system to access the memory system and to generate recollected words mentally together with working memory and word knowledge. It requires relatively restricted brain areas. The graph metrics results support the notion that the WC task necessitates the integration of widely distributed networks. Our current approach offers opportunities to address localized and global integration of domain-specific functional brain networks, as higher-order brain functions often require communication and integration between long-distant brain areas.

Third, we introduced local and global metrics for functional modules. Local metrics are calculated using all nodes within a functional module, while global metrics are computed using all nodes in the entire brain. In this study, we found that the WC task exhibited more distributed integration of whole-brain networks. Participation coefficient has been typically used to address within- and between-module interactions, and participation coefficient is calculated by a distribution of node degrees among modules or communities of a graph. In contrast, global metrics in the current study are not limited to node degrees but include more comprehensive measures, such as centralities and path lengths. Thus, our approach reveals how a functional module is integrated within the whole brain or, in other words, network communication with outside of the module in a comprehensive manner. By comparing with resting-state fMRI data and performing node randomization analyses, we validated the utility of predefined functional modules in capturing the integration of domain-specific brain networks. The language tasks exhibited distinctive patterns of graph metrics relative to the resting-state condition. Notably, while local density remained comparable, shortest path lengths and betweenness centrality differed significantly from those observed during rest. Additionally, node randomization analysis revealed that global betweenness centrality derived from the true language modules were highly distinct from the distribution of random null modules. Collectively, these results suggest that task engagement adaptively reconfigurates brain networks and confirm that predefined functional modules can effectively capture the functional integration of domain-specific brain networks.

One might ask what the differences are between BOLD activation and graph metrics. Generally speaking, BOLD-activation data are supposed to correspond to “neuronal activity level,” and network analysis is supposed to reflect interaction or dynamics of activity between regions. Change of interaction must not correspond to change of activity level of regions. In Figure 2, we observed a consistent pattern of interaction effects between the language tasks and network modules, while correlations between BOLD activation and graph metrics across subjects were quite low, as shown in Figure 5. These results suggest that within-subject relationships of BOLD activity and graph metrics between tasks or between network modules were consistent across subjects. However, BOLD activity cannot predict graph metrics based on relationships across subjects. This consistency and discrepancy suggest that graph metrics capture different aspects of brain network systems.

Regarding more technical aspects of graph theory analysis, in the current study, we employed relative thresholds to determine connections to be included in the analysis. We chose the relative threshold because of the following reasons. First, we designed this pipeline to compare task-dependent changes in brain network integration between cognitive tasks and between individuals including patient groups. In this context, global FC, the mean of correlations across whole-brain networks, could be different. Indeed, in our proof-of-concept comparison of WC and WG tasks, global FC values were significantly different. The absolute threshold method, which cut off at a particular value of the correlation coefficient, could cause a huge bias due to the differences in global FC (Alexander-Bloch et al., 2010; van den Heuvel et al., 2017). Another approach is to use weighted graph instead of choosing a particular threshold (Garrison et al., 2015). Global FC still causes systemic biases in weighted graphs. In the current study, we instead employed the most stringent approach suggested by a previous study (van den Heuvel et al., 2017), adjusting graph metrics by global FC. After correcting for global FC, we still observed consistent patterns of significant Task × Module interactions (Supporting Information Tables S5 and S6).

Head motion is known to increase spurious correlation in fMRI data (Power et al., 2014), and in the current language tasks, greater head motion was observed in the WG task than in the WC task. Therefore, one might consider that the differences in graph metrics between those tasks could be derived by the difference in head motion. Statistical values in the analysis with and without global signal scaling (Supporting Information Tables S1–S6) were almost close to each other, but sometimes those with global signal scaling were slightly lower than those without global signal scaling. These results suggest that head motion partially accounts for the difference, but the major variance was primarily explained by task demands. One might consider that the current functional modules are too small to study regional network integration. However, even the smallest network module, the motoric language module, is considerably large. The median distance between nodes in the motoric language module is 23.8 mm, and 14.6% of node pairs are more than 40 mm apart. Presumably, higher levels of functional modules have more distant node pairs. Therefore, we consider that the current approach can capture the nature of functional network integrity at multiple spatial scales.

There are several open questions and limitations in the current study. First, regarding the definition of functional modules, we primarily defined functional modules based on BAs. First, using BAs is not fully in line with functional segregation of brain networks. The boundary of BA is not always aligned with the boundary of functional parcellations. In addition, using BAs is not optimal in some cases when BAs are large, such as temporal, parietal, and medial frontal areas, and BAs are not consistent with functional segregation in some areas, such as BA8 including the lateral and medial prefrontal cortex. In fact, in this study, we split BA21 and BA22 into their anterior and posterior parts due to their functional differentiation. In contrast, while using functional parcellations seems more rational to define the boundaries of functional modules, these boundaries can be quite varied depending on the algorithm and resolution of parcellations. Another possibility for defining functional modules involves using a meta-database of functional neuroimaging data, such as Neurosynth (https://neurosynth.org/). This optimization needs to be further developed.

Second, the definition of our functional modules may not precisely correspond to the real modules in each subject and, as such, may appear arbitrary. Additionally, this relies on the debatable argument, whether those modules can be separable. However, information processing in the brain is not distinctly represented but rather gradual and distributed. Indeed, a series of studies by Romo and Salinas (2003) demonstrated that even a simple tactile discrimination task is processed in a distributed manner. While the definition and labeling of brain network modules remain a subject of debate, we argue that our multilevel spatial resolution approach can effectively capture the extent of distributed network architectures.

Lastly, in this study, we defined 1,054 nodes for the entire brain. This number is dependent on the spatial resolution of the data. Nodes can be further fractionated with the higher spatial resolution of 7T MRI data, while more aggregated with the lower spatial resolution data, such as EEG data.

## CONCLUSION

We have developed a distinctive approach that combines existing neurobiological knowledge of domain-specific brain network systems with graph network analysis. This aids in the understanding of brain network interaction, coordination, and integration. As a proof of concept, by exploiting extensive prior knowledge of language networks, we demonstrated that our approach allowed us to capture further functional properties of brain network integration. We also validated with a simple motor task to confirm our pipeline. By analyzing hierarchical networks at multiple levels and introducing local and global metrics, we have gained insights into the functional properties of brain circuits. These insights surpass those achievable through conventional regional activation analysis.

## Acknowledgments

We thank Dr. Annegret Habich for her thoughtful comments on the previous version of the manuscript. This study is supported by the Swiss National Science Foundation (192623).

## Supporting Information

Supporting information for this article is available at https://doi.org/10.1162/netn_a_00443.

## Author Contributions

Yosuke Morishima: Conceptualization; Data curation; Formal analysis; Funding acquisition; Methodology; Project administration; Software; Validation; Visualization; Writing – original draft; Writing – review & editing. Martijn van den Heuvel: Methodology; Writing – original draft; Writing – review & editing. Werner Strik: Writing – review & editing. Thomas Dierks: Conceptualization; Data curation; Formal analysis; Methodology; Writing – original draft; Writing – review & editing.

## Funding Information

“Swiss National Science Foundation (192623)”, as described in the Acknowledgments.

## Supplementary Material



## TECHNICAL TERMS

Graph network analysis: It treats brain areas as nodes, connections as edges, and uncovers connectivity patterns and integration of brain networks.
Domain-specific brain network systems: Large-scale clusters of dispersed brain regions, collectively engaged for specific cognitive tasks.
Functional module: A predefined set of brain regions, informed by prior knowledge, forming a cohesive unit locally or globally for specialized tasks.
Connectivity matrix: A matrix representing correlations between nodes, forming the basis for calculating graph-theoretical measures.
Local (graph) metrics: Measures assessing how nodes interact within a specified functional module, capturing internal connectivity and efficiency of specialized networks.
Global (graph) metrics: Measures assessing how module’s nodes interact with the entire brain, capturing overall integration beyond local module boundaries.
Node randomization: It randomly shuffles node assignments to functional modules create a null distribution, assessing significance of observed network metrics.
Relative thresholding: A fixed proportion of included connections for standardization across different global connectivity levels.
Global functional connectivity: Average of correlations across the entire brain networks, reflecting an overall level synchronization.
