# Supplementary material for: Neurobiologically informed graph theory analysis of the language system
Source: Netw Neurosci. 2025 Apr 30;9(2):504–21. doi: 10.1162/netn_a_00443 (PMC12140569; doi:10.1162/netn_a_00443)
Supplement: Supplementary file 1 [file netn-9-2-504-s001.pdf]

Supplementary Online Material for

Neurobiologically informed graph theory analysis of brain circuits

Yosuke Morishima[1], Martijn van den Heuvel[2,3], Werner Strik[1], Thomas Dierks[1]

1 Translational Research Center, University Hospital of Psychiatry and Psychotherapy, University of Bern, Bern, Switzerland

2 Department of Complex Trait Genetics, Center for Neurogenomics and Cognitive Research, Amsterdam Neuroscience, Vrije Universiteit Amsterdam, Amsterdam, The Netherlands

3 Department of Child and Adolescent Psychiatry and Psychology, Section Complex Trait Genetics, Amsterdam Neuroscience, Vrije Universiteit Medical Center, Amsterdam UMC, Amsterdam, The Netherlands

Supplementary Table 1

Task module interaction of graph metrics in different thresholds during the language tasks (no global signal scaling)

		Word recognition WC	Word recognition WG	Motoric language WC	Motoric language WG	T-value Module	p-value Module	T-value Task	p-value Task	T-value Module x Task	p-value Module x Task
20 % threshold											
	L_density	0.276	0.224	0.354	0.379	4.407	<0.0001	-2.912	0.0040	3.085	0.0024
	G_density	0.191	0.150	0.139	0.127	-5.501	<0.0001	-4.254	<0.0001	2.156	0.0324
	L_global_efficiency	0.489	0.410	0.553	0.544	3.436	0.0007	-4.196	<0.0001	2.629	0.0093
	L_nodal_efficiency	0.652	0.616	0.686	0.730	2.025	0.0444	-2.149	0.0329	3.340	0.0010
	G_nodal_efficiency	0.700	0.683	0.673	0.718	-2.084	0.0386	-1.316	0.1899	3.382	0.0009
	L_shortest_path	2.143	2.241	1.949	1.765	-2.281	0.0237	1.156	0.2493	-2.348	0.0199
	G_shortest_path	2.202	2.392	2.384	2.432	2.800	0.0057	2.921	0.0039	-1.546	0.1239
	L_clustering_coef	0.569	0.540	0.599	0.659	1.830	0.0689	-1.749	0.0820	3.822	0.0002
	G_clustering_coef	0.563	0.550	0.528	0.588	-2.821	0.0053	-1.016	0.3112	4.135	0.0001
	L_eigen_centrality	0.145	0.137	0.174	0.173	12.981	<0.0001	-3.957	0.0001	2.353	0.0197
	G_eigen_centrality	0.042	0.036	0.027	0.031	-5.575	<0.0001	-2.103	0.0369	2.351	0.0198
	L_between_centrality	26.099	26.511	17.319	13.543	-3.967	0.0001	0.186	0.8524	-1.338	0.1825
	G_between_centrality	294.703	320.426	294.616	260.185	-0.005	0.9961	1.453	0.1479	-2.403	0.0173
	assortativity	0.278	0.346	0.206	0.229	-1.917	0.0568	1.830	0.0690	-0.857	0.3923
	participation_coef	0.655	0.605	0.592	0.593	-4.325	<0.0001	-3.411	0.0008	2.489	0.0137
30 % threshold											
	L_density	0.374	0.309	0.452	0.463	4.054	0.0001	-3.395	0.0008	2.834	0.0051
	G_density	0.290	0.235	0.227	0.199	-5.428	<0.0001	-4.681	<0.0001	1.644	0.1019
	L_global_efficiency	0.614	0.535	0.651	0.630	2.218	0.0278	-4.793	<0.0001	2.475	0.0142
	L_nodal_efficiency	0.746	0.718	0.773	0.792	1.771	0.0782	-1.922	0.0562	2.248	0.0258
	G_nodal_efficiency	0.785	0.765	0.759	0.782	-2.665	0.0084	-2.111	0.0362	3.193	0.0017
	L_shortest_path	1.906	2.053	1.734	1.668	-2.504	0.0132	2.137	0.0339	-2.192	0.0297
	G_shortest_path	1.888	2.056	1.973	2.126	2.477	0.0142	4.937	<0.0001	-0.296	0.7676
	L_clustering_coef	0.646	0.622	0.682	0.724	2.321	0.0214	-1.604	0.1106	3.051	0.0026
	G_clustering_coef	0.645	0.623	0.610	0.651	-3.351	0.0010	-2.070	0.0399	4.299	<0.0001
	L_eigen_centrality	0.157	0.149	0.184	0.180	16.171	<0.0001	-4.629	<0.0001	1.752	0.0815
	G_eigen_centrality	0.047	0.041	0.034	0.035	-5.575	<0.0001	-2.368	0.0189	1.850	0.0660
	L_between_centrality	23.699	25.981	14.928	13.204	-4.870	<0.0001	1.267	0.2068	-1.573	0.1175
	G_between_centrality	232.804	255.134	211.989	222.288	-1.513	0.1320	1.623	0.1063	-0.618	0.5370
	assortativity	0.222	0.297	0.137	0.262	-2.553	0.0115	2.245	0.0260	1.066	0.2880
	participation_coef	0.740	0.690	0.707	0.695	-2.948	0.0036	-4.492	<0.0001	2.365	0.0191
40 % threshold											
	L_density	0.474	0.402	0.543	0.539	3.559	0.0005	-3.713	0.0003	2.493	0.0136
	G_density	0.391	0.327	0.326	0.279	-5.099	<0.0001	-5.005	<0.0001	0.971	0.3330
	L_global_efficiency	0.709	0.640	0.729	0.701	1.539	0.1255	-5.200	<0.0001	2.152	0.0328
	L_nodal_efficiency	0.817	0.784	0.832	0.842	1.136	0.2576	-2.550	0.0116	2.314	0.0218
	G_nodal_efficiency	0.829	0.815	0.817	0.829	-1.717	0.0877	-1.995	0.0475	2.596	0.0102
	L_shortest_path	1.685	1.806	1.547	1.565	-2.719	0.0072	2.388	0.0180	-1.437	0.1525
	G_shortest_path	1.686	1.825	1.753	1.889	2.744	<0.0001	5.652	<0.0001	-0.093	0.9260
	L_clustering_coef	0.716	0.684	0.745	0.776	1.977	0.0496	-2.211	0.0283	3.075	0.0024
	G_clustering_coef	0.696	0.677	0.674	0.701	-2.443	0.0155	-2.101	0.0370	3.603	0.0004
	L_eigen_centrality	0.165	0.159	0.190	0.185	16.836	<0.0001	-4.276	<0.0001	0.843	0.4005
	G_eigen_centrality	0.051	0.045	0.040	0.038	-5.361	<0.0001	-2.624	0.0094	1.236	0.2180
	L_between_centrality	18.997	21.403	11.595	11.805	-5.518	<0.0001	1.794	0.0745	-1.158	0.2484
	G_between_centrality	182.196	208.674	168.767	169.614	-1.373	0.1713	2.708	0.0074	-1.854	0.0654
	assortativity	0.150	0.231	0.141	0.290	-0.289	0.7727	2.599	0.0101	1.549	0.1232
	participation_coef	0.796	0.751	0.784	0.764	-1.529	0.1280	-5.488	<0.0001	2.175	0.0309

Supplementary Table 2

Task module interaction of graph metrics in different thresholds during the language tasks (incl. global signal scaling)

		Word recognition WC	Word recognition WG	Motoric language WC	Motoric language WG	T-value Module	p-value Module	T-value Task	p-value Task	T-value Module x Task	p-value Module x Task
20 % threshold											
	L_density	0.333	0.305	0.392	0.445	3.339	0.0010	-1.628	0.1052	3.299	0.0012
	G_density	0.215	0.183	0.171	0.177	-5.021	<0.0001	-3.715	0.0003	3.079	0.0024
	L_global_efficiency	0.588	0.547	0.596	0.617	0.458	0.6475	-2.263	0.0248	2.417	0.0166
	L_nodal_efficiency	0.736	0.732	0.731	0.764	-0.312	0.7555	-0.246	0.8062	1.492	0.1375
	G_nodal_efficiency	0.753	0.749	0.710	0.746	-3.805	0.0002	-0.400	0.6897	2.508	0.0130
	L_shortest_path	2.001	2.155	1.879	1.719	-1.659	0.0988	2.097	0.0374	-3.017	0.0029
	G_shortest_path	2.022	2.155	2.132	2.181	3.129	0.0020	3.777	0.0002	-1.686	0.0936
	L_clustering_coef	0.623	0.632	0.644	0.693	1.135	0.2580	0.457	0.6482	1.577	0.1165
	G_clustering_coef	0.573	0.575	0.536	0.580	-3.266	0.0013	0.124	0.9015	2.654	0.0087
	L_eigen_centrality	0.156	0.152	0.186	0.188	13.489	<0.0001	-2.002	0.0468	2.315	0.0217
	G_eigen_centrality	0.044	0.040	0.034	0.040	-3.671	0.0003	-1.557	0.1213	2.571	0.0109
	L_between_centrality	25.438	28.295	16.050	13.166	-5.223	<0.0001	1.589	0.1137	-2.258	0.0251
	G_between_centrality	244.130	263.059	277.657	258.245	1.972	0.0501	1.113	0.2670	-1.595	0.1125
	assortativity	0.292	0.358	0.232	0.292	-1.721	0.0869	1.877	0.0621	-0.112	0.9107
	participation_coef	0.737	0.705	0.693	0.720	-3.152	0.0019	-2.270	0.0244	2.968	0.0034
30 % threshold											
	L_density	0.441	0.403	0.499	0.533	3.031	0.0028	-1.968	0.0506	2.669	0.0083
	G_density	0.323	0.277	0.273	0.267	-4.577	<0.0001	-4.224	<0.0001	2.566	0.0111
	L_global_efficiency	0.689	0.657	0.698	0.700	0.589	0.5563	-2.037	0.0431	1.506	0.1339
	L_nodal_efficiency	0.807	0.799	0.808	0.831	0.071	0.9438	-0.573	0.5671	1.454	0.1477
	G_nodal_efficiency	0.797	0.798	0.771	0.797	-2.966	0.0034	0.124	0.9015	1.989	0.0483
	L_shortest_path	1.731	1.859	1.755	1.588	0.390	0.6972	2.069	0.0400	-3.371	0.0009
	G_shortest_path	1.754	1.851	1.861	1.895	3.473	0.0006	3.149	0.0019	-1.437	0.1524
	L_clustering_coef	0.686	0.685	0.723	0.760	2.267	0.0246	-0.052	0.9587	1.613	0.1086
	G_clustering_coef	0.622	0.623	0.597	0.626	-2.285	0.0235	0.013	0.9895	1.813	0.0715
	L_eigen_centrality	0.168	0.163	0.194	0.195	13.784	<0.0001	-2.514	0.0128	2.329	0.0209
	G_eigen_centrality	0.050	0.046	0.042	0.045	-3.675	0.0003	-1.826	0.0695	2.282	0.0237
	L_between_centrality	19.466	22.752	14.412	11.313	-3.633	0.0004	2.362	0.0193	-3.245	0.0014
	G_between_centrality	194.425	198.767	204.687	193.658	0.832	0.4065	0.352	0.7252	-0.881	0.3794
	assortativity	0.208	0.302	0.238	0.314	0.809	0.4198	2.499	0.0133	-0.336	0.7373
	participation_coef	0.794	0.777	0.777	0.793	-1.650	0.1006	-1.630	0.1048	2.235	0.0266
40 % threshold											
	L_density	0.544	0.504	0.591	0.606	2.555	0.0114	-2.125	0.0349	2.093	0.0378
	G_density	0.429	0.377	0.378	0.361	-4.421	<0.0001	-4.484	<0.0001	2.128	0.0347
	L_global_efficiency	0.762	0.736	0.778	0.755	1.302	0.1947	-2.058	0.0410	0.137	0.8909
	L_nodal_efficiency	0.853	0.852	0.856	0.876	0.288	0.7739	-0.150	0.8810	1.439	0.1519
	G_nodal_efficiency	0.830	0.826	0.818	0.826	-2.063	0.0406	-0.620	0.5359	1.454	0.1478
	L_shortest_path	1.516	1.587	1.532	1.442	0.413	0.6804	1.788	0.0754	-2.860	0.0047
	G_shortest_path	1.590	1.661	1.658	1.695	3.914	0.0001	4.100	0.0001	-1.369	0.1728
	L_clustering_coef	0.730	0.733	0.774	0.810	3.106	0.0022	0.215	0.8303	1.595	0.1124
	G_clustering_coef	0.665	0.659	0.654	0.664	-1.076	0.2833	-0.577	0.5645	1.136	0.2574
	L_eigen_centrality	0.176	0.173	0.201	0.201	18.655	<0.0001	-2.131	0.0345	1.166	0.2453
	G_eigen_centrality	0.054	0.050	0.047	0.049	-3.781	0.0002	-1.929	0.0553	2.022	0.0446
	L_between_centrality	13.908	15.798	10.642	8.598	-3.488	<0.0001	2.018	0.0451	-2.969	0.0034
	G_between_centrality	159.092	162.723	151.090	149.374	-1.092	0.2762	0.496	0.6207	-0.516	0.6065
	assortativity	0.145	0.195	0.174	0.324	0.726	0.4687	1.268	0.2064	1.830	0.0689
	participation_coef	0.830	0.814	0.829	0.832	-0.072	0.9428	-2.921	0.0039	2.443	0.0155

Supplementary Table 3 Statistical comparison of graph metrics of all language modules during the language tasks (no global signal scaling)

	20% Threshold				30% Threshold				40% Threshold			
	Word comprehension	Word generation	t-value	p-value	Word comprehension	Word generation	t-value	p-value	Word comprehension	Word generation	t-value	p-value
Lv4 Language-L_density	0.2133	0.1836	-5.092	<0.0001	0.3139	0.2738	-6.014	<0.0001	0.4127	0.3668	-6.521	<0.0001
Lv4 Language-G_density	0.2125	0.183	-5.092	<0.0001	0.3128	0.2728	-6.014	<0.0001	0.4112	0.3655	-6.521	<0.0001
Lv4 Language-L_global_efficiency	0.53	0.4898	-5.181	<0.0001	0.6219	0.5855	-5.949	<0.0001	0.6914	0.6576	-7.026	<0.0001
Lv4 Language-L_nodal_efficiency	0.758	0.7489	-1.7	0.0961	0.8106	0.8009	-2.599	0.0126	0.8447	0.8364	-2.499	0.0162
Lv4 Language-G_nodal_efficiency	0.758	0.7489	-1.7	0.0961	0.8106	0.8009	-2.599	0.0126	0.8447	0.8364	-2.499	0.0162
Lv4 Language-L_shortest_path_b	2.118	2.2714	4.181	0.0001	1.8319	1.9602	5.322	<0.0001	1.6489	1.7479	6.581	<0.0001
Lv4 Language-G_shortest_path_b	2.1084	2.2588	4.213	0.0001	1.8293	1.9571	5.341	<0.0001	1.6483	1.747	6.595	<0.0001
Lv4 Language-L_clustering_coef_b	0.6042	0.5985	-0.982	0.3313	0.6626	0.6539	-1.361	0.1803	0.7084	0.6972	-1.616	0.1131
Lv4 Language-G_clustering_coef_b	0.6042	0.5985	-0.982	0.3313	0.6626	0.6539	-1.361	0.1803	0.7084	0.6972	-1.616	0.1131
Lv4 Language-L_eigen_centrality_b	0.0467	0.0452	-2.986	0.0046	0.0504	0.0491	-3.67	0.0006	0.0531	0.052	-3.801	0.0004
Lv4 Language-G_eigen_centrality_b	0.0467	0.0452	-2.986	0.0046	0.0504	0.0491	-3.67	0.0006	0.0531	0.052	-3.801	0.0004
Lv4 Language-L_between_centrality_b	287.7216	320.8865	4.113	0.0002	223.5051	256.0483	5.558	<0.0001	177.3503	203.1446	6.377	<0.0001
Lv4 Language-G_between_centrality_b	287.7216	320.8865	4.113	0.0002	223.5051	256.0483	5.558	<0.0001	177.3503	203.1446	6.377	<0.0001
Lv4 Language-assortativity_b	0.2104	0.2613	2.829	0.0069	0.1836	0.2269	2.504	0.016	0.1586	0.1969	2.003	0.0512
Lv3 Language_Left-L_density	0.2563	0.2272	-2.707	0.0096	0.359	0.3223	-3.058	0.0037	0.456	0.4156	-3.329	0.0017
Lv3 Language_Left-G_density	0.411	0.3483	-4.565	<0.0001	0.5994	0.5179	-5.287	<0.0001	0.7838	0.6919	-5.842	<0.0001
Lv3 Language_Left-L_global_efficiency	0.5513	0.5145	-3.616	0.0008	0.6444	0.6088	-4.329	0.0001	0.7125	0.6805	-4.601	<0.0001
Lv3 Language_Left-L_nodal_efficiency	0.7623	0.7551	-1.088	0.2825	0.8172	0.8103	-1.312	0.1963	0.855	0.8487	-1.37	0.1776
Lv3 Language_Left-G_nodal_efficiency	0.7558	0.7476	-1.385	0.1728	0.8083	0.8001	-2.073	0.044	0.8452	0.8363	-2.39	0.0211
Lv3 Language_Left-L_shortest_path_b	2.0855	2.2135	2.707	0.0096	1.7936	1.8985	3.372	0.0015	1.6055	1.695	4.074	0.0002
Lv3 Language_Left-G_shortest_path_b	2.1144	2.2712	4.078	0.0002	1.8281	1.9609	5.182	<0.0001	1.6456	1.7495	6.476	<0.0001
Lv3 Language_Left-L_clustering_coef_b	0.6236	0.6195	-0.505	0.6161	0.6844	0.6777	-0.789	0.4343	0.7327	0.7243	-0.979	0.3328
Lv3 Language_Left-G_clustering_coef_b	0.6006	0.5958	-0.662	0.5115	0.6595	0.6507	-1.232	0.2242	0.7076	0.6956	-1.642	0.1075
Lv3 Language_Left-L_eigen_centrality_b	0.0662	0.0642	-2.763	0.0083	0.0709	0.0692	-3.269	0.0021	0.0742	0.0728	-3.068	0.0036
Lv3 Language_Left-G_eigen_centrality_b	0.0478	0.0452	-1.774	0.0828	0.0509	0.0489	-1.82	0.0755	0.0533	0.0516	-1.905	0.0632
Lv3 Language_Left-L_between_centrality_b	146.6206	161.2829	2.614	0.0121	112.1921	125.1286	3.072	0.0036	86.8556	98.8588	3.724	0.0005
Lv3 Language_Left-G_between_centrality_b	298.936	342.9802	4.107	0.0002	229.5019	265.6779	4.614	<0.0001	179.3666	210.368	5.559	<0.0001
Lv3 Language_Left-assortativity_b	0.2226	0.2608	2.195	0.0333	0.1853	0.2309	2.277	0.0276	0.1479	0.1952	2.208	0.0324
Lv3 Language_Left-participation_coef	0.3651	0.3216	-4.777	<0.0001	0.4105	0.3735	-4.362	0.0001	0.4396	0.4082	-3.985	0.0002
Lv3 Language_Right-L_density	0.2472	0.235	-1.279	0.2075	0.3548	0.3333	-1.948	0.0577	0.4572	0.4313	-2.224	0.0312
Lv3 Language_Right-G_density	0.4397	0.3855	-4.107	0.0002	0.6538	0.5764	-4.967	<0.0001	0.8648	0.7746	-5.313	<0.0001
Lv3 Language_Right-L_global_efficiency	0.5435	0.5122	-2.888	0.0059	0.6389	0.6119	-3.004	0.0043	0.7108	0.6879	-3.112	0.0032
Lv3 Language_Right-L_nodal_efficiency	0.7577	0.7522	-0.765	0.4483	0.8159	0.807	-1.558	0.1263	0.8503	0.8455	-1.106	0.2745
Lv3 Language_Right-G_nodal_efficiency	0.7605	0.7503	-1.598	0.1171	0.8131	0.8018	-2.32	0.0249	0.8441	0.8364	-2.11	0.0404
Lv3 Language_Right-L_shortest_path_b	2.0586	2.127	1.792	0.0799	1.7827	1.846	2.445	0.0184	1.6069	1.6612	2.512	0.0157
Lv3 Language_Right-G_shortest_path_b	2.1016	2.2449	4.06	0.0002	1.8306	1.953	5.011	<0.0001	1.6513	1.7443	5.795	<0.0001
Lv3 Language_Right-L_clustering_coef_b	0.6145	0.6202	0.797	0.4294	0.678	0.679	0.126	0.9002	0.7259	0.7247	-0.154	0.878
Lv3 Language_Right-G_clustering_coef_b	0.6082	0.6016	-1.132	0.2637	0.6659	0.6575	-1.257	0.2153	0.7093	0.6991	-1.398	0.1688
Lv3 Language_Right-L_eigen_centrality_b	0.0705	0.0688	-1.976	0.0543	0.0753	0.0738	-2.138	0.038	0.0786	0.0776	-2.075	0.0437
Lv3 Language_Right-G_eigen_centrality_b	0.0454	0.0452	-0.172	0.8639	0.0498	0.0492	-0.459	0.6487	0.0529	0.0523	-0.549	0.5857
Lv3 Language_Right-L_between_centrality_b	126.5337	129.6907	0.718	0.4766	98.1444	104.0551	1.891	0.0651	77.7139	83.7853	2.229	0.0309
Lv3 Language_Right-G_between_centrality_b	275.223	296.263	2.177	0.0347	216.8216	245.3162	3.811	0.0004	175.1032	195.094	3.873	0.0003
Lv3 Language_Right-assortativity_b	0.2046	0.2487	1.924	0.0606	0.1769	0.1958	0.859	0.3946	0.1389	0.1495	0.521	0.6047
Lv3 Language_Right-participation_coef	0.3918	0.3517	-4.055	0.0002	0.4314	0.401	-3.655	0.0007	0.4566	0.4336	-3.401	0.0014
Lv2 Word processing_Left-L_density	0.2338	0.1955	-3.007	0.0043	0.3354	0.2856	-3.401	0.0014	0.4384	0.3812	-3.645	0.0007
Lv2 Word processing_Left-G_density	0.771	0.6078	-4.503	<0.0001	1.184	0.9603	-5.226	<0.0001	1.6088	1.3466	-5.738	<0.0001
Lv2 Word processing_Left-L_global_efficiency	0.5067	0.4505	-3.763	0.0005	0.6184	0.5632	-4.614	<0.0001	0.697	0.6484	-4.839	<0.0001
Lv2 Word processing_Left-L_nodal_efficiency	0.7018	0.673	-2.257	0.0289	0.7818	0.7605	-2.002	0.0513	0.8353	0.8118	-3.103	0.0033
Lv2 Word processing_Left-G_nodal_efficiency	0.7194	0.6913	-2.595	0.0127	0.7884	0.7705	-2.62	0.0119	0.8342	0.8188	-2.889	0.0059
Lv2 Word processing_Left-L_shortest_path_b	2.2384	2.4272	2.863	0.0063	1.8968	2.0489	3.207	0.0025	1.6576	1.8022	4.714	<0.0001
Lv2 Word processing_Left-G_shortest_path_b	2.2167	2.431	4.244	0.0001	1.9024	2.0679	4.992	<0.0001	1.7038	1.8323	6.22	<0.0001
Lv2 Word processing_Left-L_clustering_coef_b	0.5805	0.5591	-1.834	0.0732	0.6525	0.6348	-1.517	0.1362	0.7101	0.6882	-2.037	0.0476
Lv2 Word processing_Left-G_clustering_coef_b	0.5739	0.5537	-1.945	0.058	0.6419	0.6245	-2.001	0.0515	0.6941	0.6761	-2.091	0.0422
Lv2 Word processing_Left-L_eigen_centrality_b	0.0966	0.092	-3.217	0.0024	0.105	0.101	-3.123	0.0031	0.1112	0.1076	-3.456	0.0012
Lv2 Word processing_Left-G_eigen_centrality_b	0.0376	0.033	-2.295	0.0265	0.0428	0.0382	-2.737	0.0088	0.0469	0.0427	-3.088	0.0034
Lv2 Word processing_Left-L_between_centrality_b	69.1664	76.2745	2.057	0.0455	54.5788	61.3872	2.561	0.0139	40.8967	48.998	4.159	0.0001
Lv2 Word processing_Left-G_between_centrality_b	301.3733	358.0595	3.329	0.0017	237.4675	271.4823	3.367	0.0016	185.6915	216.2577	4.457	0.0001
Lv2 Word processing_Left-assortativity_b	0.2208	0.2866	2.543	0.0145	0.1665	0.2439	2.93	0.0053	0.116	0.1903	2.856	0.0065
Lv2 Word processing_Left-participation_coef	0.5692	0.5036	-5.839	<0.0001	0.6504	0.5958	-6.083	<0.0001	0.7037	0.6604	-6.51	<0.0001
Lv2 Word processing_Right-L_density	0.2566	0.2267	-2.239	0.0301	0.3688	0.3226	-2.864	0.0063	0.4764	0.426	-2.9	0.0058
Lv2 Word processing_Right-G_density	0.9838	0.7944	-5.114	<0.0001	1.5163	1.2426	-6.142	<0.0001	2.0551	1.7311	-6.57	<0.0001
Lv2 Word processing_Right-L_global_efficiency	0.5196	0.4667	-3.097	0.0034	0.6349	0.5862	-3.462	0.0012	0.7158	0.6743	-3.648	0.0007
Lv2 Word processing_Right-L_nodal_efficiency	0.7044	0.6787	-1.745	0.0878	0.791	0.7642	-2.477	0.0171	0.8397	0.8237	-2.07	0.0442
Lv2 Word processing_Right-G_nodal_efficiency	0.726	0.7019	-1.99	0.0527	0.7947	0.7756	-2.394	0.0209	0.8325	0.8201	-2.73	0.009
Lv2 Word processing_Right-L_shortest_path_b	2.1085	2.217	1.77	0.0835	1.8104	1.9318	2.749	0.0086	1.5933	1.7148	3.565	0.0009
Lv2 Word processing_Right-G_shortest_path_b	2.1508	2.3509	4.449	0.0001	1.8615	2.0207	4.793	<0.0001	1.6689	1.8007	6.549	<0.0001
Lv2 Word processing_Right-L_clustering_coef_b	0.5835	0.5719	-0.826	0.4131	0.6634	0.6463	-1.466	0.1496	0.7205	0.7081	-1.166	0.2498
Lv2 Word processing_Right-G_clustering_coef_b	0.5781	0.5615	-1.647	0.1066	0.6443	0.6322	-1.463	0.1505	0.692	0.6793	-1.491	0.143
Lv2 Word processing_Right-L_eigen_centrality_b	0.1082	0.1054	-1.83	0.0739	0.1172	0.1139	-2.397	0.0208	0.1235	0.1204	-2.751	0.0085
Lv2 Word processing_Right-G_eigen_centrality_b	0.04	0.0367	-2.109	0.0406	0.0459	0.0419	-3.108	0.0033	0.0502	0.0463	-3.636	0.0007
Lv2 Word processing_Right-L_between_centrality_b	50.9155	52.0894	0.36	0.7206	40.0476	44.8187	2.158	0.0363	30.1854	35.8179	3.21	0.0024
Lv2 Word processing_Right-G_between_centrality_b	290.2456	304.4253	1.013	0.3164	236.6451	256.684	2.438	0.0188	193.5611	206.3439	1.891	0.0651
Lv2 Word processing_Right-assortativity_b	0.2337	0.2406	0.246	0.8072	0.1789	0.2127	1.27	0.2105	0.1198	0.1758	1.922	0.0609
Lv2 Word processing_Right-participation_coef	0.6096	0.5485	-4.869	<0.0001	0.6807	0.6337	-5.003	<0.0001	0.7255	0.688	-6.311	<0.0001
Lv2 Language Sensory_Left-L_density	0.4996	0.4861	-0.764	0.4491	0.6099	0.6083	-0.096	0.924	0.6909	0.703	0.805	0.4253
Lv2 Language Sensory_Left-G_density	1.3566	1.1852	-4.41	0.0001	1.8629	1.6776	-4.553	<0.0001	2.3123	2.141	-4.294	0.0001
Lv2 Language Sensory_Left-L_global_efficiency	0.7334	0.7265	-0.697	0.4896	0.7979	0.7987	0.093	0.9264	0.8427	0.8494	0.872	0.3877
Lv2 Language Sensory_Left-L_nodal_efficiency	0.8771	0.8747	-0.517	0.6074	0.9034	0.9026	-0.201	0.8414	0.9223	0.9233	0.252	0.8022
Lv2 Language Sensory_Left-G_nodal_efficiency	0.8255	0.8192	-1.386	0.1725	0.8487	0.8391	-2.073	0.0439	0.8669	0.8564	-2.312	0.0254
Lv2 Language Sensory_Left-L_shortest_path_b	1.5986	1.6133	0.545	0.5885	1.4302	1.4249	-0.253	0.8011	1.3259	1.3099	-0.966	0.3394
Lv2 Language Sensory_Left-G_shortest_path_b	1.9274	2.0455	4.711	<0.0001	1.6914	1.7814	5.525	<0.0001	1.5424	1.6066	5.435	<0.0001
Lv2 Language Sensory_Left-L_clustering_coef_b	0.7615	0.7571	-0.499	0.6201	0.8086	0.8065	-0.251	0.8027	0.8449	0.8468	0.243	0.8093
Lv2 Language Sensory_Left-G_clustering_coef_b	0.6563	0.644	-1.329	0.1906	0.698	0.6794	-2.017	0.0496	0.7342	0.7132	-2.294	0.0265
Lv2 Language Sensory_Left-L_eigen_centrality_b	0.1189	0.1195	0.699	0.4879	0.1225	0.1235	1.42	0.1624	0.1248	0.1257	1.906	0.063
Lv2 Language Sensory_Left-G_eigen_centrality_b	0.0667	0.0644	-1.356	0.182	0.0663	0.0661	-0.155	0.8776	0.0653	0.0665	1.105	0.2749
Lv2 Language Sensory_Left-L_between_centrality_b	34.661	35.5284	0.556	0.581	24.9344	24.647	-0.239	0.8121	18.9042	17.9757	-0.966	0.3394

Lv2 Language Sensory_Left-G_between_centrality_b	303.3298	358.8918	3.511	0.001	228.7193	278.9058	4.283	0.0001	178.6766	220.8072	4.516	<0.0001
Lv2 Language Sensory_Left-assortativity_b	0.1686	0.1324	-1.598	0.117	0.145	0.0772	-2.864	0.0063	0.1159	0.0392	-2.974	0.0047
Lv2 Language Sensory_Left-participation_coef	0.6873	0.6319	-6.389	<0.0001	0.7251	0.687	-6.431	<0.0001	0.7463	0.7198	-6.042	<0.0001
Lv2 Language Sensory_Right-L_density	0.4225	0.4424	1.126	0.2661	0.5396	0.5665	1.423	0.1615	0.6346	0.6675	1.784	0.0812
Lv2 Language Sensory_Right-G_density	1.2155	1.1074	-2.712	0.0094	1.7218	1.5888	-2.869	0.0063	2.1901	2.0563	-2.719	0.0093
Lv2 Language Sensory_Right-L_global_efficiency	0.6843	0.6953	0.977	0.3336	0.759	0.7733	1.344	0.1857	0.8121	0.8305	1.883	0.0661
Lv2 Language Sensory_Right-L_nodal_efficiency	0.857	0.8658	1.707	0.0947	0.8823	0.8958	2.617	0.012	0.9056	0.9166	2.149	0.037
Lv2 Language Sensory_Right-G_nodal_efficiency	0.8294	0.8239	-1.218	0.2294	0.8502	0.845	-1.187	0.2414	0.8684	0.8615	-1.653	0.1052
Lv2 Language Sensory_Right-L_shortest_path_b	1.712	1.7108	-0.028	0.9779	1.5289	1.4886	-1.397	0.1694	1.3976	1.3523	-2.021	0.0493
Lv2 Language Sensory_Right-G_shortest_path_b	1.9948	2.1004	3.41	0.0014	1.7441	1.822	4.084	0.0002	1.5832	1.6384	3.967	0.0003
Lv2 Language Sensory_Right-L_clustering_coef_b	0.7257	0.7461	2.127	0.0389	0.7708	0.7949	2.382	0.0215	0.8133	0.8349	2.147	0.0372
Lv2 Language Sensory_Right-G_clustering_coef_b	0.6665	0.6578	-1.026	0.3103	0.7036	0.693	-1.218	0.2296	0.7381	0.7245	-1.617	0.1129
Lv2 Language Sensory_Right-L_eigen_centrality_b	0.118	0.1179	-0.174	0.8626	0.1222	0.1227	0.568	0.5731	0.1249	0.1256	1.12	0.2686
Lv2 Language Sensory_Right-G_eigen_centrality_b	0.0574	0.0588	0.507	0.6145	0.0596	0.0614	0.888	0.3794	0.0604	0.0627	1.424	0.1612
Lv2 Language Sensory_Right-L_between_centrality_b	40.2909	40.2616	-0.012	0.9901	30.1484	27.7991	-1.424	0.1613	22.6612	20.0832	-2.021	0.0493
Lv2 Language Sensory_Right-G_between_centrality_b	243.6368	297.0502	3.497	0.0011	193.7371	239.615	4.232	0.0001	154.5639	191.1415	5.268	<0.0001
Lv2 Language Sensory_Right-assortativity_b	0.1899	0.1865	-0.149	0.8819	0.1585	0.1217	-1.292	0.2028	0.1302	0.0693	-1.923	0.0609
Lv2 Language Sensory_Right-participation_coef	0.6702	0.6214	-5.918	<0.0001	0.7124	0.6752	-5.831	<0.0001	0.7371	0.7113	-5.764	<0.0001
Lv2 Language Motor_Left-L_density	0.3556	0.3803	1.342	0.1862	0.4513	0.4624	0.596	0.554	0.5408	0.5393	-0.084	0.9334
Lv2 Language Motor_Left-G_density	1.6659	1.5279	-1.084	0.2841	2.728	2.3955	-2.109	0.0406	3.9122	3.3634	-3.149	0.0029
Lv2 Language Motor_Left-L_global_efficiency	0.557	0.5464	-0.588	0.5597	0.6521	0.6315	-1.322	0.1928	0.7275	0.7016	-2.01	0.0504
Lv2 Language Motor_Left-L_nodal_efficiency	0.6935	0.734	2.372	0.022	0.7686	0.7847	1.151	0.2559	0.8279	0.8419	1.13	0.2643
Lv2 Language Motor_Left-G_nodal_efficiency	0.6784	0.721	3.377	0.0015	0.7602	0.7823	2.372	0.022	0.8199	0.8337	2.072	0.044
Lv2 Language Motor_Left-L_shortest_path_b	1.8965	1.7654	-2.076	0.0436	1.7404	1.6542	-1.474	0.1476	1.5588	1.5665	0.145	0.8856
Lv2 Language Motor_Left-G_shortest_path_b	2.3431	2.4332	1.21	0.2328	1.9788	2.133	3.163	0.0028	1.7505	1.8884	4.743	<0.0001
Lv2 Language Motor_Left-L_clustering_coef_b	0.6066	0.6635	3.293	0.0019	0.6789	0.7155	2.501	0.0161	0.742	0.7757	2.431	0.0191
Lv2 Language Motor_Left-G_clustering_coef_b	0.5321	0.5891	4.974	<0.0001	0.61	0.65	4.436	0.0001	0.6772	0.7043	3.348	0.0017
Lv2 Language Motor_Left-L_eigen_centrality_b	0.1745	0.1727	-0.929	0.3578	0.1831	0.1803	-1.836	0.073	0.1897	0.186	-2.777	0.008
Lv2 Language Motor_Left-G_eigen_centrality_b	0.0272	0.0301	0.96	0.3423	0.0339	0.0345	0.235	0.8149	0.0399	0.0385	-0.623	0.5366
Lv2 Language Motor_Left-L_between_centrality_b	16.4272	13.5879	-2.022	0.0492	15.0265	12.9395	-1.707	0.0947	11.8469	11.8544	0.006	0.995
Lv2 Language Motor_Left-G_between_centrality_b	280.8829	260.2039	-1.108	0.2738	209.3445	215.594	0.442	0.6606	163.5367	167.2006	0.38	0.7058
Lv2 Language Motor_Left-assortativity_b	0.1858	0.2408	1.726	0.0912	0.1478	0.2591	3.185	0.0026	0.1425	0.2822	4.64	<0.0001
Lv2 Language Motor_Left-participation_coef	0.5554	0.5553	-0.006	0.9951	0.657	0.6471	-0.725	0.472	0.728	0.7075	-2.439	0.0187
Lv2 Language Motor_Right-L_density	0.2899	0.3092	1.096	0.279	0.3916	0.3939	0.126	0.9001	0.4881	0.4759	-0.623	0.5363
Lv2 Language Motor_Right-G_density	1.7327	1.6492	-0.699	0.4884	2.9232	2.642	-1.897	0.0642	4.2513	3.7826	-2.79	0.0077
Lv2 Language Motor_Right-L_global_efficiency	0.4746	0.4501	-1.175	0.246	0.5956	0.5494	-2.408	0.0202	0.689	0.656	-1.822	0.075
Lv2 Language Motor_Right-L_nodal_efficiency	0.6238	0.6418	0.793	0.4317	0.7257	0.7154	-0.522	0.6042	0.7934	0.7834	-0.581	0.5642
Lv2 Language Motor_Right-G_nodal_efficiency	0.6521	0.6649	0.596	0.5544	0.754	0.7461	-0.552	0.5838	0.8047	0.8069	0.189	0.8512
Lv2 Language Motor_Right-L_shortest_path_b	1.9233	1.7334	-2.577	0.0133	1.8119	1.6575	-2.134	0.0383	1.6392	1.6514	0.224	0.8236
Lv2 Language Motor_Right-G_shortest_path_b	2.3003	2.4107	1.901	0.0637	2.0074	2.1624	4.022	0.0002	1.8048	1.9051	3.005	0.0043
Lv2 Language Motor_Right-L_clustering_coef_b	0.5469	0.58	1.547	0.1289	0.6445	0.6516	0.376	0.7089	0.7099	0.7159	0.365	0.717
Lv2 Language Motor_Right-G_clustering_coef_b	0.5192	0.5451	1.326	0.1914	0.6139	0.6212	0.556	0.5812	0.6719	0.6779	0.512	0.6112
Lv2 Language Motor_Right-L_eigen_centrality_b	0.1785	0.1774	-0.399	0.6921	0.1897	0.1871	-1.143	0.2591	0.1985	0.1959	-1.279	0.2076
Lv2 Language Motor_Right-G_eigen_centrality_b	0.025	0.0283	1.375	0.176	0.0319	0.0334	0.721	0.4748	0.038	0.0382	0.082	0.9352
Lv2 Language Motor_Right-L_between_centrality_b	13.0478	9.6478	-2.372	0.022	13.813	9.9457	-2.648	0.0111	11.4283	11.3891	-0.037	0.9707
Lv2 Language Motor_Right-G_between_centrality_b	327.0132	272.3499	-1.653	0.1053	231.2346	231.725	0.029	0.9768	185.7539	176.7441	-0.738	0.4643
Lv2 Language Motor_Right-assortativity_b	0.1598	0.2299	1.639	0.1081	0.1556	0.2612	2.651	0.011	0.1533	0.2244	1.853	0.0705
Lv2 Language Motor_Right-participation_coef	0.5478	0.5377	-0.569	0.5724	0.6524	0.639	-0.892	0.377	0.7181	0.7063	-1.087	0.283
Lv1 Word Recognition_Left-L_density	0.2777	0.2264	-3.359	0.0016	0.3785	0.3124	-4.05	0.0002	0.4786	0.4047	-4.158	0.0001
Lv1 Word Recognition_Left-G_density	1.8352	1.4404	-4.495	<0.0001	2.7936	2.2529	-5.304	<0.0001	3.7601	3.1265	-5.832	<0.0001
Lv1 Word Recognition_Left-L_global_efficiency	0.4905	0.4163	-3.799	0.0004	0.6184	0.5419	-4.871	<0.0001	0.7099	0.6455	-5.314	<0.0001
Lv1 Word Recognition_Left-L_nodal_efficiency	0.6578	0.6206	-2.046	0.0466	0.7462	0.7252	-1.235	0.2232	0.8193	0.7878	-2.096	0.0418
Lv1 Word Recognition_Left-G_nodal_efficiency	0.7064	0.6849	-1.662	0.1036	0.7799	0.7676	-1.206	0.2341	0.8314	0.8176	-1.579	0.1214
Lv1 Word Recognition_Left-L_shortest_path_b	2.1592	2.2477	0.89	0.3784	1.8882	2.0499	2.7	0.0097	1.6683	1.8307	3.873	0.0003
Lv1 Word Recognition_Left-G_shortest_path_b	2.1912	2.4062	3.995	0.0002	1.888	2.056	5.132	<0.0001	1.6845	1.8288	6.795	<0.0001
Lv1 Word Recognition_Left-L_clustering_coef_b	0.5698	0.543	-1.591	0.1187	0.648	0.6295	-1.138	0.2612	0.7184	0.6883	-1.902	0.0636
Lv1 Word Recognition_Left-G_clustering_coef_b	0.5664	0.549	-1.322	0.1927	0.64	0.626	-1.238	0.2223	0.6952	0.6789	-1.477	0.1467
Lv1 Word Recognition_Left-L_eigen_centrality_b	0.1459	0.1375	-3.997	0.0002	0.1571	0.1495	-4.737	<0.0001	0.1657	0.1596	-4.476	0.0001
Lv1 Word Recognition_Left-G_eigen_centrality_b	0.042	0.0363	-2.634	0.0115	0.047	0.0414	-3.247	0.0022	0.0508	0.0456	-3.758	0.0005
Lv1 Word Recognition_Left-L_between_centrality_b	26.8276	27.1289	0.105	0.9165	23.1649	25.9805	1.603	0.116	18.4363	22.2504	3.036	0.004
Lv1 Word Recognition_Left-G_between_centrality_b	289.2107	336.1082	2.117	0.0398	236.2197	256.8761	1.768	0.0839	184.6067	211.5817	3.476	0.0011
Lv1 Word Recognition_Left-assortativity_b	0.269	0.3589	2.611	0.0122	0.2236	0.2941	2.028	0.0485	0.1554	0.2228	2.167	0.0356
Lv1 Word Recognition_Left-participation_coef	0.6571	0.605	-4.278	0.0001	0.739	0.6873	-5.255	<0.0001	0.7905	0.7499	-5.252	<0.0001
Lv1 Word Recognition_Right-L_density	0.306	0.2599	-2.92	0.0054	0.4141	0.3537	-3.275	0.002	0.5171	0.4466	-3.524	0.001
Lv1 Word Recognition_Right-G_density	1.8794	1.4966	-4.767	<0.0001	2.8727	2.3303	-5.477	<0.0001	3.8631	3.2275	-5.773	<0.0001
Lv1 Word Recognition_Right-L_global_efficiency	0.5343	0.4556	-3.914	0.0003	0.6469	0.582	-3.896	0.0003	0.7301	0.6698	-4.313	0.0001
Lv1 Word Recognition_Right-L_nodal_efficiency	0.7066	0.6669	-2.242	0.0299	0.787	0.7549	-2.186	0.034	0.8363	0.8172	-1.756	0.0858
Lv1 Word Recognition_Right-G_nodal_efficiency	0.7238	0.7022	-1.854	0.0703	0.7977	0.7753	-2.507	0.0158	0.8311	0.8179	-2.215	0.0319
Lv1 Word Recognition_Right-L_shortest_path_b	2.0413	2.0853	0.549	0.5859	1.755	1.9367	3.079	0.0035	1.5619	1.7159	3.356	0.0016
Lv1 Word Recognition_Right-G_shortest_path_b	2.1249	2.3321	4.898	<0.0001	1.8393	2.012	5.618	<0.0001	1.651	1.7987	6.982	<0.0001
Lv1 Word Recognition_Right-L_clustering_coef_b	0.6093	0.5787	-1.851	0.0707	0.6804	0.6593	-1.361	0.1804	0.7329	0.718	-1.165	0.25
Lv1 Word Recognition_Right-G_clustering_coef_b	0.5774	0.5603	-1.567	0.1242	0.6493	0.6344	-1.363	0.1795	0.6942	0.6812	-1.275	0.2088
Lv1 Word Recognition_Right-L_eigen_centrality_b	0.1503	0.1445	-2.832	0.0069	0.1608	0.1552	-3.238	0.0023	0.1687	0.1631	-3.846	0.0004
Lv1 Word Recognition_Right-G_eigen_centrality_b	0.0421	0.0371	-2.517	0.0155	0.048	0.0427	-3.243	0.0022	0.0521	0.0472	-3.674	0.0006
Lv1 Word Recognition_Right-L_between_centrality_b	25.4603	23.2849	-0.928	0.3586	20.0465	23.7931	2.303	0.026	15.3583	19.1034	2.945	0.0051
Lv1 Word Recognition_Right-G_between_centrality_b	269.5121	297.1991	1.639	0.1082	217.9075	243.2671	2.445	0.0185	182.7514	195.7394	1.378	0.1749
Lv1 Word Recognition_Right-assortativity_b	0.2554	0.26	0.135	0.8932	0.1881	0.2284	1.4	0.1684	0.1326	0.2165	2.849	0.0066
Lv1 Word Recognition_Right-participation_coef	0.6759	0.6162	-4.401	0.0001	0.7521	0.702	-4.987	<0.0001	0.7961	0.7564	-5.276	<0.0001
Lv1 Semantic Processing_Left-L_density	0.2765	0.244	-2.403	0.0204	0.3819	0.3431	-2.479	0.017	0.4844	0.4419	-2.536	0.0147
Lv1 Semantic Processing_Left-G_density	1.3182	1.0434	-4.052	0.0002	2.0408	1.6641	-4.489	<0.0001	2.7977	2.3561	-4.843	<0.0001
Lv1 Semantic Processing_Left-L_global_efficiency	0.541	0.4844	-3.364	0.0016	0.6418	0.5971	-3.159	0.0028	0.719	0.6769	-3.628	0.0007
Lv1 Semantic Processing_Left-L_nodal_efficiency	0.7097	0.6586	-3.096	0.0034	0.7906	0.7574	-2.532	0.0149	0.8429	0.8139	-3.251	0.0022
Lv1 Semantic Processing_Left-G_nodal_efficiency	0.7302	0.6966	-2.479	0.017	0.7955	0.7729	-2.872	0.0062	0.8365	0.8198	-2.983	0.0046
Lv1 Semantic Processing_Left-L_shortest_path_b	2.1013	2.1929	1.224	0.2273	1.802	1.894	2.069	0.0443	1.5965	1.6876	2.792	0.0077
Lv1 Semantic Processing_Left-G_shortest_path_b	2.2365	2.4463	3.681	0.0006	1.9146	2.077	4.451	0.0001	1.72	1.8358	5.065	<0.0001
Lv1 Semantic Processing_Left-L_clustering_coef_b	0.5961	0.5585	-2.659	0.0108	0.6665	0.6449	-1.607	0.1151	0.7258	0.7019	-2.197	0.0332
Lv1 Semantic Processing_Left-G_clustering_coef_b	0.5801	0.5576	-1.875	0.0672	0.6435	0.6233	-2.123	0.0393	0.6932	0.6738	-2.118	0.0398
Lv1 Semantic Processing_Left-L_eigen_centrality_b	0.1375	0.1311	-2.783	0.0079	0.1473	0.1425	-2.681	0.0102	0.1543	0.1495	-3.834	0.0004
Lv1 Semantic Processing_Left-G_eigen_centrality_b	0.0341	0.0302	-1.704	0.0953	0.0393	0.0355	-1.934	0.0594	0.0437	0.0403	-2.036	0.0476
Lv1 Semantic Processing_Left-L_between_centrality_b	33.5478	34.4149	0.386	0.7013	25.8199	27.9503	1.504	0.1397	19.8298	22.1665	2.096	0.0417
Lv1 Semantic Processing_Left-G_between_centrality_b	311.4509	376.2477	2.637	0.0114	238.5013	283.5846	3.278	0.002	186.5903	220.1321	3.723	0.0005
Lv1 Semantic Processing_Left-assortativity_b	0.1466	0.2251	2.367	0.0223	0.0944	0.1894	3.085	0.0035	0.055	0.1465	3.725	0.0005
Lv1 Semantic Processing_Left-participation_coef	0.6564	0.5915	-4.734	<0.0001	0.7398	0.691	-4.633	<0.0001	0.7922	0.7548	-5.368	<0.0001
Lv1 Semantic Processing_Right-L_density	0.3298	0.328	-0.096	0.9243	0.4447	0.4374	-0.361	0.7197	0.5525	0.5465	-0.314	0.7546
Lv1 Semantic Processing_Right-G_density	2.0539	1.6888	-4.255	0.0001	3.2003	2.6573	-5.21	<0.0001	4.3817	3.7297	-5.504	<0.0001
Lv1 Semantic Processing_Right-L_global_efficiency	0.5641	0.5286	-1.625	0.1111	0.6721	0.6522	-1.167	0.2494	0.7552	0.7417	-1.079	0.2864
Lv1 Semantic Processing_Right-L_nodal_efficiency	0.7108	0.6803	-1.538	0.131	0.792	0.7763	-0.995	0.3252	0.853	0.8539	0.084	0.9338
Lv1 Semantic Processing_Right-G_nodal_efficiency	0.7287	0.7015	-1.592	0.1185	0.7911	0.7759	-1.317	0.1944	0.8341	0.8227	-2.209	0.0323
Lv1 Semantic Processing_Right-L_shortest_path_b	1.92	1.9106	-0.165	0.87	1.6937	1.6997	0.126	0.9004	1.5099	1.5148	0.153	0.8792
Lv1 Semantic Processing_Right-G_shortest_path_b	2.1794	2.3693	3.531	0.001	1.8877	2.0302	3.419	0.0013	1.6902	1.8034	4.439	0.0001

Lv1 Semantic Processing_Right-L_clustering_coef_b	0.6105	0.5991	-0.639	0.5262	0.6843	0.6853	0.066	0.9478	0.7518	0.7631	0.884	0.3813
Lv1 Semantic Processing_Right-G_clustering_coef_b	0.5789	0.5629	-1.146	0.258	0.6383	0.6295	-0.876	0.3856	0.6894	0.6771	-1.491	0.1429
Lv1 Semantic Processing_Right-L_eigen_centrality_b	0.1709	0.1671	-1.545	0.1293	0.1825	0.1784	-2.276	0.0276	0.1893	0.1865	-2.003	0.0513
Lv1 Semantic Processing_Right-G_eigen_centrality_b	0.0375	0.0361	-0.734	0.4668	0.0433	0.0408	-1.487	0.1441	0.0478	0.0452	-1.721	0.0922
Lv1 Semantic Processing_Right-L_between_centrality_	18.8207	17.2917	-1.043	0.3026	14.8641	14.846	-0.018	0.9858	11.4656	11.3967	-0.095	0.9248
Lv1 Semantic Processing_Right-G_between_centrality_	315.2986	313.157	-0.103	0.9185	259.2864	272.896	0.875	0.3865	206.6229	219.1576	1.348	0.1844
Lv1 Semantic Processing_Right-assortativity_b	0.1021	0.1733	2.604	0.0124	0.0511	0.1527	3.408	0.0014	0.0293	0.1029	2.697	0.0098
Lv1 Semantic Processing_Right-participation_coef	0.7016	0.6462	-3.321	0.0018	0.7711	0.734	-2.682	0.0102	0.8148	0.7901	-3.069	0.0036
Lv1 Syntatic Processing_Left-L_density	0.5254	0.5204	-0.257	0.7986	0.6309	0.642	0.608	0.5461	0.7084	0.7343	1.535	0.1318
Lv1 Syntatic Processing_Left-G_density	1.5343	1.3329	-4.398	0.0001	2.1016	1.891	-4.38	0.0001	2.605	2.4151	-4.036	0.0002
Lv1 Syntatic Processing_Left-L_global_efficiency	0.7499	0.7462	-0.337	0.738	0.8103	0.8166	0.64	0.5252	0.8521	0.8654	1.522	0.135
Lv1 Syntatic Processing_Left-L_nodal_efficiency	0.8808	0.8809	0.014	0.9887	0.9063	0.9079	0.359	0.7216	0.9246	0.9285	0.956	0.3443
Lv1 Syntatic Processing_Left-G_nodal_efficiency	0.8261	0.8188	-1.479	0.1462	0.8491	0.8393	-2.017	0.0497	0.8681	0.8569	-2.271	0.028
Lv1 Syntatic Processing_Left-L_shortest_path_b	1.5535	1.5631	0.312	0.7562	1.4002	1.385	-0.664	0.5098	1.3042	1.2762	-1.469	0.1487
Lv1 Syntatic Processing_Left-G_shortest_path_b	1.9282	2.05	5.054	<0.0001	1.6944	1.7852	5.692	<0.0001	1.5467	1.6098	5.236	<0.0001
Lv1 Syntatic Processing_Left-L_clustering_coef_b	0.7672	0.7684	0.119	0.9058	0.8134	0.8174	0.449	0.6558	0.8494	0.8578	1.017	0.3144
Lv1 Syntatic Processing_Left-G_clustering_coef_b	0.656	0.6431	-1.325	0.1918	0.6987	0.6797	-1.957	0.0566	0.7363	0.7141	-2.255	0.0291
Lv1 Syntatic Processing_Left-L_eigen_centrality_b	0.1286	0.1284	-0.171	0.8652	0.1319	0.1326	0.95	0.3471	0.1338	0.1347	1.557	0.1266
Lv1 Syntatic Processing_Left-G_eigen_centrality_b	0.0661	0.0634	-1.458	0.1517	0.0657	0.0655	-0.162	0.872	0.0648	0.066	1.064	0.2932
Lv1 Syntatic Processing_Left-L_between_centrality_b	28.2274	28.6856	0.294	0.7705	20.408	19.6338	-0.664	0.5098	15.5134	14.0844	-1.469	0.1487
Lv1 Syntatic Processing_Left-G_between_centrality_b	292.9085	352.0877	3.461	0.0012	217.465	274.0695	4.545	<0.0001	168.3736	217.0465	4.866	<0.0001
Lv1 Syntatic Processing_Left-assortativity_b	0.1342	0.108	-1.119	0.2689	0.1069	0.0455	-2.773	0.008	0.0676	0.012	-2.342	0.0237
Lv1 Syntatic Processing_Left-participation_coef	0.7337	0.6673	-7.257	<0.0001	0.7765	0.729	-7.534	<0.0001	0.8	0.7663	-7.154	<0.0001
Lv1 Syntatic Processing_Right-L_density	0.4833	0.5238	1.831	0.0737	0.5926	0.6512	2.574	0.0134	0.6776	0.7496	3.285	0.002
Lv1 Syntatic Processing_Right-G_density	1.4525	1.325	-2.336	0.024	2.0366	1.8961	-2.241	0.03	2.5759	2.4479	-1.925	0.0606
Lv1 Syntatic Processing_Right-L_global_efficiency	0.7261	0.7488	1.713	0.0936	0.7897	0.822	2.591	0.0128	0.8358	0.8738	3.323	0.0018
Lv1 Syntatic Processing_Right-L_nodal_efficiency	0.8677	0.8799	2.144	0.0375	0.8926	0.9084	2.794	0.0076	0.9129	0.9305	3.179	0.0027
Lv1 Syntatic Processing_Right-G_nodal_efficiency	0.8291	0.8262	-0.595	0.5546	0.8503	0.8447	-1.121	0.2683	0.8683	0.861	-1.547	0.1287
Lv1 Syntatic Processing_Right-L_shortest_path_b	1.6109	1.5562	-1.455	0.1526	1.4476	1.3707	-2.573	0.0135	1.3412	1.2563	-3.359	0.0016
Lv1 Syntatic Processing_Right-G_shortest_path_b	1.9837	2.0913	3.819	0.0004	1.7417	1.8164	3.803	0.0004	1.5856	1.6341	3.168	0.0028
Lv1 Syntatic Processing_Right-L_clustering_coef_b	0.7422	0.766	2.248	0.0295	0.7883	0.8181	2.727	0.0091	0.827	0.8612	3.11	0.0032
Lv1 Syntatic Processing_Right-G_clustering_coef_b	0.6625	0.6566	-0.614	0.542	0.7019	0.6907	-1.153	0.2551	0.7373	0.7226	-1.57	0.1235
Lv1 Syntatic Processing_Right-L_eigen_centrality_b	0.1316	0.1326	0.938	0.3533	0.1353	0.1367	1.694	0.0972	0.1376	0.1391	2.328	0.0245
Lv1 Syntatic Processing_Right-G_eigen_centrality_b	0.0573	0.0588	0.493	0.6247	0.0591	0.0616	1.009	0.3182	0.0597	0.0629	1.622	0.1118
Lv1 Syntatic Processing_Right-L_between_centrality_b	29.2981	26.6539	-1.473	0.1478	21.4862	17.7924	-2.573	0.0135	16.3789	12.3017	-3.359	0.0016
Lv1 Syntatic Processing_Right-G_between_centrality_b	247.2894	305.5907	3.397	0.0014	189.5892	246.0708	4.422	0.0001	151.7141	195.813	5.253	<0.0001
Lv1 Syntatic Processing_Right-assortativity_b	0.1581	0.1021	-2.332	0.0242	0.1103	0.0427	-2.675	0.0104	0.0731	-0.0049	-3.005	0.0043
Lv1 Syntatic Processing_Right-participation_coef	0.7092	0.6474	-6.472	<0.0001	0.7578	0.7102	-6.53	<0.0001	0.7863	0.752	-6.316	<0.0001
Lv1 Acoustic Processing_Left-L_density	0.8613	0.8623	0.038	0.9697	0.9172	0.9161	-0.051	0.9593	0.9534	0.9513	-0.156	0.8764
Lv1 Acoustic Processing_Left-G_density	11.7063	10.642	-1.896	0.0644	16.3705	14.827	-2.63	0.0116	20.5115	18.8199	-2.884	0.006
Lv1 Acoustic Processing_Left-L_global_efficiency	0.9193	0.9166	-0.13	0.8973	0.9551	0.9567	0.127	0.8995	0.9767	0.9757	-0.156	0.8764
Lv1 Acoustic Processing_Left-L_nodal_efficiency	0.9246	0.9307	0.301	0.7645	0.9568	0.9674	1.191	0.2399	0.9774	0.9805	0.559	0.5788
Lv1 Acoustic Processing_Left-G_nodal_efficiency	0.821	0.8218	0.121	0.9045	0.8454	0.8382	-1.393	0.1703	0.8587	0.853	-1.296	0.2015
Lv1 Acoustic Processing_Left-L_shortest_path_b	1.1449	1.1297	-0.54	0.5921	1.0807	1.0921	0.5	0.6198	1.0466	1.0487	0.156	0.8764
Lv1 Acoustic Processing_Left-G_shortest_path_b	1.9252	2.0115	1.89	0.0652	1.6696	1.753	2.907	0.0056	1.5103	1.5824	3.659	0.0007
Lv1 Acoustic Processing_Left-L_clustering_coef_b	0.898	0.9051	0.372	0.7117	0.9367	0.9475	0.907	0.3691	0.9654	0.9652	-0.026	0.9793
Lv1 Acoustic Processing_Left-G_clustering_coef_b	0.6583	0.6505	-0.696	0.4901	0.6924	0.6776	-1.44	0.1567	0.7189	0.7064	-1.43	0.1596
Lv1 Acoustic Processing_Left-L_eigen_centrality_b	0.3711	0.3697	-0.507	0.6148	0.3746	0.3745	-0.064	0.9493	0.3762	0.3764	0.357	0.7228
Lv1 Acoustic Processing_Left-G_eigen_centrality_b	0.0713	0.0717	0.094	0.9256	0.0708	0.0708	0.002	0.9984	0.0693	0.07	0.299	0.7663
Lv1 Acoustic Processing_Left-L_between_centrality_b	0.795	0.7578	-0.256	0.7991	0.472	0.5528	0.599	0.5524	0.2795	0.2919	0.156	0.8764
Lv1 Acoustic Processing_Left-G_between_centrality_b	380.7452	409.4362	0.639	0.526	312.3229	314.8327	0.068	0.9465	255.2135	248.7438	-0.215	0.8309
Lv1 Acoustic Processing_Left-participation_coef	0.8188	0.8166	-0.231	0.8183	0.8367	0.8369	0.038	0.97	0.8492	0.8474	-0.423	0.674
Lv1 Acoustic Processing_Right-L_density	0.7723	0.7802	0.344	0.7325	0.8587	0.8424	-0.832	0.4096	0.907	0.8913	-0.912	0.3667
Lv1 Acoustic Processing_Right-G_density	7.423	6.716	-2.302	0.026	11.1371	9.7813	-3.724	0.0005	14.602	12.8408	-4.579	<0.0001
Lv1 Acoustic Processing_Right-L_global_efficiency	0.8605	0.8593	-0.058	0.9544	0.9204	0.9084	-0.914	0.3658	0.9506	0.9442	-0.676	0.5025
Lv1 Acoustic Processing_Right-L_nodal_efficiency	0.8991	0.8997	0.038	0.9698	0.9393	0.9482	0.937	0.3537	0.9636	0.9587	-0.479	0.6342
Lv1 Acoustic Processing_Right-G_nodal_efficiency	0.8305	0.8117	-1.714	0.0934	0.8494	0.8467	-0.461	0.6471	0.8693	0.8644	-1.093	0.2803
Lv1 Acoustic Processing_Right-L_shortest_path_b	1.221	1.2165	-0.114	0.9099	1.1531	1.1624	0.391	0.6977	1.1147	1.1171	0.094	0.9257
Lv1 Acoustic Processing_Right-G_shortest_path_b	2.0576	2.1545	1.328	0.1908	1.7571	1.8534	2.929	0.0053	1.5702	1.6615	4.435	0.0001
Lv1 Acoustic Processing_Right-L_clustering_coef_b	0.8509	0.8658	0.898	0.3737	0.9031	0.9165	1.227	0.2263	0.9416	0.9298	-1.002	0.3217
Lv1 Acoustic Processing_Right-G_clustering_coef_b	0.688	0.6639	-1.832	0.0735	0.7125	0.706	-0.696	0.4897	0.7422	0.7351	-0.922	0.3615
Lv1 Acoustic Processing_Right-L_eigen_centrality_b	0.3216	0.3212	-0.168	0.8672	0.3269	0.3276	0.433	0.6669	0.3293	0.3299	0.539	0.5925
Lv1 Acoustic Processing_Right-G_eigen_centrality_b	0.0581	0.0583	0.043	0.9655	0.0623	0.0605	-0.723	0.4735	0.0642	0.062	-1.182	0.2436
Lv1 Acoustic Processing_Right-L_between_centrality_b	1.6087	1.5942	-0.047	0.9625	1.1884	1.2657	0.403	0.6891	0.9179	0.9372	0.094	0.9257
Lv1 Acoustic Processing_Right-G_between_centrality_l	223.7503	250.5521	1.349	0.1842	216.3197	204.4668	-0.619	0.5389	170.0792	165.7076	-0.35	0.7283
Lv1 Acoustic Processing_Right-participation_coef	0.7818	0.7802	-0.109	0.9139	0.8247	0.8185	-0.592	0.5569	0.8425	0.8417	-0.14	0.8889
Lv1 Motoric Language_Left-L_density	0.3556	0.3803	1.342	0.1862	0.4513	0.4624	0.596	0.554	0.5408	0.5393	-0.084	0.9334
Lv1 Motoric Language_Left-G_density	1.6659	1.5279	-1.084	0.2841	2.728	2.3955	-2.109	0.0406	3.9122	3.3634	-3.149	0.0029
Lv1 Motoric Language_Left-L_global_efficiency	0.557	0.5464	-0.588	0.5597	0.6521	0.6315	-1.322	0.1928	0.7275	0.7016	-2.01	0.0504
Lv1 Motoric Language_Left-L_nodal_efficiency	0.6935	0.734	2.372	0.022	0.7686	0.7847	1.151	0.2559	0.8279	0.8419	1.13	0.2643
Lv1 Motoric Language_Left-G_nodal_efficiency	0.6784	0.721	3.377	0.0015	0.7602	0.7823	2.372	0.022	0.8199	0.8337	2.072	0.044
Lv1 Motoric Language_Left-L_shortest_path_b	1.8965	1.7654	-2.076	0.0436	1.7404	1.6542	-1.474	0.1476	1.5588	1.5665	0.145	0.8856
Lv1 Motoric Language_Left-G_shortest_path_b	2.3431	2.4332	1.21	0.2328	1.9788	2.133	3.163	0.0028	1.7505	1.8884	4.743	<0.0001
Lv1 Motoric Language_Left-L_clustering_coef_b	0.6066	0.6635	3.293	0.0019	0.6789	0.7155	2.501	0.0161	0.742	0.7757	2.431	0.0191
Lv1 Motoric Language_Left-G_clustering_coef_b	0.5321	0.5891	4.974	<0.0001	0.61	0.65	4.436	0.0001	0.6772	0.7043	3.348	0.0017
Lv1 Motoric Language_Left-L_eigen_centrality_b	0.1745	0.1727	-0.929	0.3578	0.1831	0.1803	-1.836	0.073	0.1897	0.186	-2.777	0.008
Lv1 Motoric Language_Left-G_eigen_centrality_b	0.0272	0.0301	0.96	0.3423	0.0339	0.0345	0.235	0.8149	0.0399	0.0385	-0.623	0.5366
Lv1 Motoric Language_Left-L_between_centrality_b	16.4272	13.5879	-2.022	0.0492	15.0265	12.9395	-1.707	0.0947	11.8469	11.8544	0.006	0.995
Lv1 Motoric Language_Left-G_between_centrality_b	280.8829	260.2039	-1.108	0.2738	209.3445	215.594	0.442	0.6606	163.5367	167.2006	0.38	0.7058
Lv1 Motoric Language_Left-assortativity_b	0.1858	0.2408	1.726	0.0912	0.1478	0.2591	3.185	0.0026	0.1425	0.2822	4.64	<0.0001
Lv1 Motoric Language_Left-participation_coef	0.5874	0.5896	0.129	0.8977	0.6995	0.6917	-0.546	0.5876	0.7785	0.7584	-2.349	0.0233
Lv1 Motoric Language_Right-L_density	0.2899	0.3092	1.096	0.279	0.3916	0.3939	0.126	0.9001	0.4881	0.4759	-0.623	0.5363
Lv1 Motoric Language_Right-G_density	1.7327	1.6492	-0.699	0.4884	2.9232	2.642	-1.897	0.0642	4.2513	3.7826	-2.79	0.0077
Lv1 Motoric Language_Right-L_global_efficiency	0.4746	0.4501	-1.175	0.246	0.5956	0.5494	-2.408	0.0202	0.689	0.656	-1.822	0.075
Lv1 Motoric Language_Right-L_nodal_efficiency	0.6238	0.6418	0.793	0.4317	0.7257	0.7154	-0.522	0.6042	0.7934	0.7834	-0.581	0.5642
Lv1 Motoric Language_Right-G_nodal_efficiency	0.6521	0.6649	0.596	0.5544	0.754	0.7461	-0.552	0.5838	0.8047	0.8069	0.189	0.8512
Lv1 Motoric Language_Right-L_shortest_path_b	1.9233	1.7334	-2.577	0.0133	1.8119	1.6575	-2.134	0.0383	1.6392	1.6514	0.224	0.8236
Lv1 Motoric Language_Right-G_shortest_path_b	2.3003	2.4107	1.901	0.0637	2.0074	2.1624	4.022	0.0002	1.8048	1.9051	3.005	0.0043
Lv1 Motoric Language_Right-L_clustering_coef_b	0.5469	0.58	1.547	0.1289	0.6445	0.6516	0.376	0.7089	0.7099	0.7159	0.365	0.717
Lv1 Motoric Language_Right-G_clustering_coef_b	0.5192	0.5451	1.326	0.1914	0.6139	0.6212	0.556	0.5812	0.6719	0.6779	0.512	0.6112
Lv1 Motoric Language_Right-L_eigen_centrality_b	0.1785	0.1774	-0.399	0.6921	0.1897	0.1871	-1.143	0.2591	0.1985	0.1959	-1.279	0.2076
Lv1 Motoric Language_Right-G_eigen_centrality_b	0.025	0.0283	1.375	0.176	0.0319	0.0334	0.721	0.4748	0.038	0.0382	0.082	0.9352
Lv1 Motoric Language_Right-L_between_centrality_b	13.0478	9.6478	-2.372	0.022	13.813	9.9457	-2.648	0.0111	11.4283	11.3891	-0.037	0.9707
Lv1 Motoric Language_Right-G_between_centrality_b	327.0132	272.3499	-1.653	0.1053	231.2346	231.725	0.029	0.9768	185.7539	176.7441	-0.738	0.4643
Lv1 Motoric Language_Right-assortativity_b	0.1598	0.2299	1.639	0.1081	0.1556	0.2612	2.651	0.011	0.1533	0.2244	1.853	0.0705
Lv1 Motoric Language_Right-participation_coef	0.5769	0.569	-0.417	0.6788	0.6914	0.6783	-0.819	0.4171	0.7649	0.7538	-0.979	0.333

Supplementary Table 4: Statistical comparison of graph metrics of all language modules during the language tasks (incl. global signal scaling)

	20% Threshold				30% Threshold				40% Threshold			
	Word comprehension	Word generation	t-value	p-value	Word comprehension	Word generation	t-value	p-value	Word comprehension	Word generation	t-value	p-value
Lv4 Language-L_density	0.2375	0.2144	-3.9	0.0003	0.3427	0.3108	-4.853	<0.0001	0.4433	0.4078	-5.287	<0.0001
Lv4 Language-G_density	0.2366	0.2136	-3.9	0.0003	0.3414	0.3097	-4.853	<0.0001	0.4417	0.4063	-5.287	<0.0001
Lv4 Language-L_global_efficiency	0.5794	0.5585	-2.909	0.0056	0.6589	0.6386	-3.834	0.0004	0.7185	0.6987	-4.857	<0.0001
Lv4 Language-L_nodal_efficiency	0.776	0.7813	1.302	0.1995	0.8118	0.8108	-0.316	0.7531	0.8371	0.8317	-1.518	<0.0001
Lv4 Language-G_nodal_efficiency	0.776	0.7813	1.302	0.1995	0.8118	0.8108	-0.316	0.7531	0.8371	0.8317	-1.518	0.136
Lv4 Language-L_shortest_path_b	1.9834	2.0673	2.501	0.0161	1.7301	1.7897	2.744	0.0087	1.575	1.6226	4.026	0.0002
Lv4 Language-G_shortest_path_b	1.9804	2.065	2.605	0.0124	1.7296	1.7896	2.786	0.0078	1.575	1.6226	4.026	0.0002
Lv4 Language-L_clustering_coef_b	0.5932	0.5989	0.819	0.4168	0.6381	0.6331	-0.67	0.5065	0.6777	0.667	-1.449	0.1544
Lv4 Language-G_clustering_coef_b	0.5932	0.5989	0.819	0.4168	0.6381	0.6331	-0.67	0.5065	0.6777	0.667	-1.449	0.1544
Lv4 Language-L_eigen_centrality_b	0.05	0.0483	-2.784	0.0078	0.0537	0.0525	-2.446	0.0184	0.056	0.0554	-1.666	0.1027
Lv4 Language-G_eigen_centrality_b	0.05	0.0483	-2.784	0.0078	0.0537	0.0525	-2.446	0.0184	0.056	0.0554	-1.666	0.1027
Lv4 Language-L_between_centrality_b	260.463	284.2814	3.274	0.002	196.4572	212.8805	2.948	0.0051	155.1859	167.9942	4.055	0.0002
Lv4 Language-G_between_centrality_b	260.463	284.2814	3.274	0.002	196.4572	212.8805	2.948	0.0051	155.1859	167.9942	4.055	0.0002
Lv3 Language_Left-L_density	0.2738	0.2717	-0.223	0.8249	0.3832	0.3755	-0.723	0.4733	0.4855	0.4754	-0.941	0.3519
Lv3 Language_Left-G_density	0.2309	0.2122	-2.978	0.0047	0.3356	0.3081	-3.908	0.0003	0.4365	0.4049	-4.344	0.0001
Lv3 Language_Left-L_global_efficiency	0.5915	0.5842	-0.805	0.4248	0.6749	0.6698	-0.709	0.4823	0.738	0.7319	-1.049	0.2999
Lv3 Language_Left-L_nodal_efficiency	0.781	0.789	1.484	0.1449	0.8188	0.826	1.642	0.1076	0.8482	0.8523	1.009	0.3184
Lv3 Language_Left-G_nodal_efficiency	0.7744	0.7797	1.086	0.2831	0.8096	0.8109	0.371	0.7124	0.8363	0.8324	-1.061	0.2942
Lv3 Language_Left-L_shortest_path_b	1.9483	1.9922	1.337	0.188	1.7048	1.7344	1.187	0.2413	1.542	1.5614	1.271	0.2104
Lv3 Language_Left-G_shortest_path_b	1.9847	2.0684	2.774	0.008	1.7399	1.7948	2.413	0.02	1.5818	1.625	3.525	0.001
Lv3 Language_Left-L_clustering_coef_b	0.6133	0.6334	2.688	0.01	0.6605	0.6734	1.521	0.1353	0.704	0.7115	0.875	0.3865
Lv3 Language_Left-G_clustering_coef_b	0.5931	0.6007	1.083	0.2846	0.6372	0.6354	-0.242	0.8095	0.6775	0.6689	-1.089	0.2819
Lv3 Language_Left-L_eigen_centrality_b	0.0735	0.0711	-3.134	0.003	0.0778	0.0766	-2.148	0.0372	0.0806	0.08	-1.109	0.2732
Lv3 Language_Left-G_eigen_centrality_b	0.0485	0.048	-0.317	0.7526	0.0528	0.0523	-0.429	0.6703	0.0554	0.0553	-0.211	0.8342
Lv3 Language_Left-L_between_centrality_b	121.4358	127.4132	1.512	0.1375	92.1667	96.6728	1.484	0.1447	71.4884	74.1023	1.303	0.1994
Lv3 Language_Left-G_between_centrality_b	250.6981	270.6667	2.882	0.006	191.421	206.0062	2.457	0.0179	152.3672	164.5141	3.462	0.0012
Lv3 Language_Left-assortativity_b	0.2414	0.307	3.353	0.0016	0.2034	0.2497	2.315	0.0252	0.1644	0.1903	1.303	0.1991
Lv3 Language_Left-participation_coef	0.423	0.3952	-2.716	0.0093	0.4545	0.4342	-2.351	0.0232	0.4729	0.4573	-2.255	0.0291
Lv3 Language_Right-L_density	0.293	0.2728	-1.851	0.0708	0.4025	0.377	-2.135	0.0382	0.5024	0.4758	-2.252	0.0293
Lv3 Language_Right-G_density	0.2421	0.2149	-3.882	0.0003	0.347	0.3112	-4.724	<0.0001	0.4467	0.4076	-5.211	<0.0001
Lv3 Language_Right-L_global_efficiency	0.6111	0.5934	-2.01	0.0505	0.69	0.6728	-2.344	0.0235	0.7481	0.7327	-2.437	0.0188
Lv3 Language_Right-L_nodal_efficiency	0.7928	0.7987	1.127	0.2656	0.833	0.8304	-0.554	0.5822	0.8571	0.8526	-0.96	0.342
Lv3 Language_Right-G_nodal_efficiency	0.7775	0.7829	1.295	0.2019	0.814	0.8106	-0.896	0.3751	0.8378	0.8311	-1.809	0.0772
Lv3 Language_Right-L_shortest_path_b	1.9252	1.9812	1.379	0.1748	1.6639	1.7128	2.112	0.0402	1.5142	1.5536	2.541	0.0146
Lv3 Language_Right-G_shortest_path_b	1.9759	2.0617	2.364	0.0224	1.7196	1.7847	3.034	0.004	1.5684	1.6202	4.195	0.0001
Lv3 Language_Right-L_clustering_coef_b	0.6301	0.6346	0.509	0.613	0.6794	0.6735	-0.63	0.532	0.7178	0.7091	-0.943	0.3506
Lv3 Language_Right-G_clustering_coef_b	0.5932	0.5972	0.51	0.6125	0.6389	0.6309	-1.005	0.3204	0.678	0.6651	-1.697	0.0966
Lv3 Language_Right-L_eigen_centrality_b	0.0724	0.0699	-3.113	0.0032	0.0768	0.0751	-2.725	0.0091	0.0796	0.0786	-2.061	0.0451
Lv3 Language_Right-G_eigen_centrality_b	0.0514	0.0485	-2.042	0.047	0.0546	0.0527	-1.761	0.0851	0.0565	0.0555	-1.359	0.181
Lv3 Language_Right-L_between_centrality_b	125.2949	133.0274	1.56	0.1257	90.8188	97.4052	2.098	0.0416	70.4074	75.7706	2.519	0.0154
Lv3 Language_Right-G_between_centrality_b	269.8741	297.4028	2.852	0.0065	201.311	219.5057	2.558	0.014	157.9025	171.3483	2.973	0.0047
Lv3 Language_Right-assortativity_b	0.227	0.3122	4.265	0.0001	0.1918	0.2655	3.425	0.0013	0.1505	0.2157	3.055	0.0038
Lv3 Language_Right-participation_coef	0.4095	0.3771	-3.126	0.0031	0.4473	0.4191	-3.084	0.0035	0.4668	0.4451	-2.789	0.0077
Lv2 Word processing_Left-L_density	0.2946	0.2881	-0.448	0.6566	0.4118	0.3974	-0.848	0.4011	0.5245	0.5061	-1.015	0.3154
Lv2 Word processing_Left-G_density	0.2114	0.1852	-3.621	0.0007	0.3191	0.2801	-4.458	0.0001	0.4256	0.3802	-4.807	<0.0001
Lv2 Word processing_Left-L_global_efficiency	0.5886	0.5711	-1.141	0.2601	0.6837	0.6703	-1.039	0.3044	0.7557	0.743	-1.209	0.2328
Lv2 Word processing_Left-L_nodal_efficiency	0.7525	0.7568	0.306	0.7607	0.8124	0.8142	0.171	0.8649	0.8532	0.8563	0.507	0.6149
Lv2 Word processing_Left-G_nodal_efficiency	0.7543	0.7568	0.237	0.8134	0.7975	0.7998	0.398	0.6925	0.8281	0.8257	-0.566	0.5744
Lv2 Word processing_Left-L_shortest_path_b	2.0048	2.0932	1.426	0.1607	1.7006	1.7615	1.452	0.1535	1.5099	1.5538	1.6	0.1167
Lv2 Word processing_Left-G_shortest_path_b	2.0265	2.1425	2.887	0.006	1.7621	1.8381	2.562	0.0138	1.594	1.6539	3.662	0.0007
Lv2 Word processing_Left-L_clustering_coef_b	0.6057	0.6241	1.362	0.18	0.6666	0.6763	0.788	0.4346	0.7186	0.7264	0.707	0.4829
Lv2 Word processing_Left-G_clustering_coef_b	0.5701	0.5785	0.871	0.3883	0.6191	0.6207	0.177	0.8603	0.6613	0.657	-0.484	0.6305
Lv2 Word processing_Left-L_eigen_centrality_b	0.1161	0.1142	-1.161	0.2518	0.1248	0.1225	-1.722	0.0919	0.1304	0.1284	-1.871	0.0678
Lv2 Word processing_Left-G_eigen_centrality_b	0.0426	0.0408	-0.923	0.3609	0.049	0.0465	-1.828	0.0742	0.0533	0.051	-2.202	0.0328
Lv2 Word processing_Left-L_between_centrality_b	47.6939	51.8866	1.61	0.1145	34.4876	37.6462	1.665	0.1028	25.4356	27.6633	1.64	0.1079
Lv2 Word processing_Left-G_between_centrality_b	267.5159	277.1839	1.104	0.2755	205.9035	208.9804	0.476	0.6364	165.3983	167.9927	0.577	0.5667
Lv2 Word processing_Left-assortativity_b	0.2383	0.2982	2.209	0.0323	0.1756	0.2332	2.028	0.0485	0.1155	0.1678	1.799	0.0788
Lv2 Word processing_Left-participation_coef	0.672	0.6381	-2.611	0.0122	0.728	0.7071	-2.196	0.0333	0.762	0.7454	-3.043	0.0039
Lv2 Word processing_Right-L_density	0.2949	0.275	-1.371	0.177	0.4104	0.3867	-1.461	0.1509	0.521	0.4932	-1.687	0.0985
Lv2 Word processing_Right-G_density	0.2088	0.1833	-3.037	0.004	0.3134	0.2773	-3.781	0.0005	0.4176	0.3755	-4.259	0.0001
Lv2 Word processing_Right-L_global_efficiency	0.5948	0.5722	-1.611	0.1141	0.686	0.6686	-1.564	0.1247	0.7541	0.7367	-1.818	0.0757
Lv2 Word processing_Right-L_nodal_efficiency	0.7615	0.7697	0.812	0.4212	0.8213	0.8217	0.058	0.9537	0.8559	0.8529	-0.57	0.5718
Lv2 Word processing_Right-G_nodal_efficiency	0.7561	0.765	1.444	0.1557	0.8037	0.8034	-0.071	0.9438	0.8311	0.827	-1.129	0.2649
Lv2 Word processing_Right-L_shortest_path_b	2.0222	2.0565	0.536	0.5946	1.7091	1.7513	1.104	0.2756	1.5134	1.5611	1.91	0.0626
Lv2 Word processing_Right-G_shortest_path_b	2.0488	2.1498	2.283	0.0272	1.7692	1.8413	2.603	0.0125	1.6022	1.661	3.548	0.0009
Lv2 Word processing_Right-L_clustering_coef_b	0.6168	0.6235	0.628	0.533	0.6752	0.675	-0.026	0.9797	0.7221	0.7172	-0.521	0.6048
Lv2 Word processing_Right-G_clustering_coef_b	0.5766	0.5854	1.029	0.3091	0.6266	0.6238	-0.339	0.7361	0.6666	0.6595	-0.962	0.3411
Lv2 Word processing_Right-L_eigen_centrality_b	0.1056	0.1033	-1.471	0.1482	0.1133	0.1114	-1.605	0.1156	0.1184	0.1168	-1.905	0.0632
Lv2 Word processing_Right-G_eigen_centrality_b	0.0422	0.04	-1.017	0.3147	0.0479	0.0457	-1.344	0.1857	0.0518	0.05	-1.411	0.1651
Lv2 Word processing_Right-L_between_centrality_b	60.4979	61.5722	0.324	0.7476	43.1241	45.4741	1.022	0.3121	31.2735	34.1115	1.869	0.0681
Lv2 Word processing_Right-G_between_centrality_b	264.9836	283.8742	1.899	0.064	198.3322	208.1214	1.463	0.1504	157.0908	164.2072	1.631	0.1098
Lv2 Word processing_Right-assortativity_b	0.2154	0.285	2.903	0.0057	0.167	0.2254	2.729	0.009	0.1169	0.1802	3.026	0.0041
Lv2 Word processing_Right-participation_coef	0.6518	0.6248	-2.324	0.0247	0.7192	0.6937	-2.898	0.0058	0.7549	0.7365	-3.193	0.0026
Lv2 Language Sensory_Left-L_density	0.4274	0.4492	1.408	0.1659	0.5357	0.5618	1.614	0.1136	0.6218	0.6545	2.069	0.0443
Lv2 Language Sensory_Left-G_density	0.2676	0.2469	-2.774	0.008	0.3708	0.3456	-3.119	0.0032	0.4657	0.4407	-3.071	0.0036
Lv2 Language Sensory_Left-L_global_efficiency	0.6949	0.7064	1.257	0.2152	0.7615	0.7753	1.606	0.1153	0.8086	0.8256	2.076	0.0436
Lv2 Language Sensory_Left-L_nodal_efficiency	0.8531	0.866	2.612	0.0122	0.8758	0.888	2.226	0.0311	0.8945	0.9066	2.278	0.0275
Lv2 Language Sensory_Left-G_nodal_efficiency	0.8133	0.8107	-0.619	0.5389	0.8328	0.825	-1.731	0.0902	0.8494	0.8401	-2.158	0.0363
Lv2 Language Sensory_Left-L_shortest_path_b	1.6866	1.6637	-0.872	0.3878	1.5029	1.4718	-1.513	0.1372	1.3918	1.3556	-2.054	0.0458
Lv2 Language Sensory_Left-G_shortest_path_b	1.9008	1.9691	2.784	0.0078	1.6802	1.7241	2.937	0.0052	1.5454	1.5766	3.28	0.002
Lv2 Language Sensory_Left-L_clustering_coef_b	0.7135	0.7394	2.657	0.0109	0.7529	0.7774	2.264	0.0285	0.7894	0.8134	2.262	0.0286
Lv2 Language Sensory_Left-G_clustering_coef_b	0.6321	0.6262	-0.648	0.5201	0.6662	0.651	-1.681	0.0998	0.6989	0.6805	-2.15	0.037
Lv2 Language Sensory_Left-L_eigen_centrality_b	0.1177	0.1158	-2.388	0.0212	0.1212	0.1203	-1.373	0.1765	0.1231	0.1229	-0.284	0.7777
Lv2 Language Sensory_Left-G_eigen_centrality_b	0.0584	0.0569	-0.61	0.545	0.0597	0.0597	-0.015	0.988	0.0601	0.0611	0.773	0.4434
Lv2 Language Sensory_Left-L_between_centrality_b	41.114	39.8225	-0.825	0.4137	30.1761	28.3108	-1.513	0.1372	23.5103	21.3357	-2.054	0.0458
Lv2 Language Sensory_Left-G_between_centrality_b	227.3563	269.4942	4.676	<0.0001	174.7456	207.7705	4.893	<0.0001	141.9122	166.8178	4.822	<0.0001

Lv2 Language Sensory_Left-assortativity_b	0.206	0.2408	1.461	0.1509	0.1657	0.1708	0.18	0.8581	0.1249	0.1064	-0.651	0.5185
Lv2 Language Sensory_Left-participation_coef	0.7037	0.6632	-5.453	<0.0001	0.7396	0.7114	-5.042	<0.0001	0.7621	0.741	-4.987	<0.0001
Lv2 Language Sensory_Right-L_density	0.5101	0.4887	-1.326	0.1915	0.6129	0.6037	-0.577	0.5668	0.6848	0.6942	0.62	0.5381
Lv2 Language Sensory_Right-G_density	0.3001	0.2612	-5.297	<0.0001	0.4051	0.3634	-5.492	<0.0001	0.4967	0.4602	-4.868	<0.0001
Lv2 Language Sensory_Right-L_global_efficiency	0.7433	0.734	-1.081	0.2855	0.8023	0.799	-0.407	0.6861	0.8411	0.8463	0.684	0.4977
Lv2 Language Sensory_Right-L_nodal_efficiency	0.8729	0.8689	-0.825	0.4136	0.8965	0.8918	-0.946	0.3491	0.9131	0.9123	-0.155	0.8774
Lv2 Language Sensory_Right-G_nodal_efficiency	0.8097	0.8007	-1.944	0.0581	0.8303	0.8168	-2.802	0.0075	0.8493	0.8337	-3.432	0.0013
Lv2 Language Sensory_Right-L_shortest_path_b	1.5616	1.5742	0.617	0.5405	1.4119	1.4136	0.092	0.9269	1.3232	1.3107	-0.801	0.4273
Lv2 Language Sensory_Right-G_shortest_path_b	1.8482	1.9316	4.156	0.0001	1.6382	1.6968	4.666	<0.0001	1.5124	1.5541	4.92	<0.0001
Lv2 Language Sensory_Right-L_clustering_coef_b	0.75	0.7421	-0.831	0.4104	0.7947	0.7845	-1.03	0.3083	0.8266	0.825	-0.17	0.8655
Lv2 Language Sensory_Right-G_clustering_coef_b	0.6225	0.6048	-1.811	0.0768	0.661	0.6343	-2.773	0.008	0.6987	0.6676	-3.418	0.0013
Lv2 Language Sensory_Right-L_eigen_centrality_b	0.1252	0.1249	-0.433	0.6669	0.1283	0.1288	0.926	0.3592	0.1301	0.131	2.065	0.0447
Lv2 Language Sensory_Right-G_eigen_centrality_b	0.0675	0.0611	-3.69	0.0006	0.0662	0.0636	-2.036	0.0477	0.0647	0.0643	-0.362	0.7188
Lv2 Language Sensory_Right-L_between_centrality_b	30.3265	31.0079	0.617	0.5405	22.2443	22.3328	0.092	0.9269	17.4545	16.7763	-0.801	0.4273
Lv2 Language Sensory_Right-G_between_centrality_b	281.5432	329.0021	3.014	0.0042	207.3403	244.3694	3.38	0.0015	159.0691	190.6268	4.175	0.0001
Lv2 Language Sensory_Right-assortativity_b	0.1729	0.1709	-0.079	0.9377	0.1542	0.1021	-2.218	0.0317	0.1222	0.0459	-3.312	0.0018
Lv2 Language Sensory_Right-participation_coef	0.7201	0.6784	-7.641	<0.0001	0.7521	0.7242	-7.015	<0.0001	0.7704	0.7514	-6.082	<0.0001
Lv2 Language Motor_Left-L_density	0.3917	0.4449	3.169	0.0027	0.4986	0.5331	1.907	0.0629	0.591	0.6064	0.931	0.357
Lv2 Language Motor_Left-G_density	0.1714	0.177	0.631	0.5313	0.2735	0.2671	-0.631	0.5313	0.378	0.3609	-1.61	0.1144
Lv2 Language Motor_Left-L_global_efficiency	0.596	0.6168	1.143	0.2591	0.6983	0.6997	0.082	0.9347	0.7775	0.7546	-1.765	0.0844
Lv2 Language Motor_Left-L_nodal_efficiency	0.7307	0.7644	2.059	0.0453	0.8085	0.8308	1.625	0.1112	0.8564	0.8759	2.157	0.0364
Lv2 Language Motor_Left-G_nodal_efficiency	0.7099	0.7456	2.822	0.0071	0.7713	0.7971	2.373	0.022	0.8181	0.8261	1.182	0.2434
Lv2 Language Motor_Left-L_shortest_path_b	1.8785	1.7189	-2.119	0.0396	1.7548	1.5877	-2.484	0.0168	1.5321	1.4422	-2.055	0.0457
Lv2 Language Motor_Left-G_shortest_path_b	2.1319	2.181	1.258	0.215	1.8607	1.8949	0.878	0.3848	1.6578	1.6955	1.846	0.0715
Lv2 Language Motor_Left-L_clustering_coef_b	0.644	0.6932	2.87	0.0062	0.7232	0.7596	2.801	0.0075	0.7743	0.8098	3.06	0.0037
Lv2 Language Motor_Left-G_clustering_coef_b	0.536	0.5803	3.209	0.0025	0.597	0.6257	2.226	0.0311	0.6544	0.6643	0.899	0.3734
Lv2 Language Motor_Left-L_eigen_centrality_b	0.1856	0.1884	1.502	0.1401	0.194	0.1955	0.814	0.4197	0.2013	0.2006	-0.518	0.6067
Lv2 Language Motor_Left-G_eigen_centrality_b	0.0342	0.0395	2.321	0.0249	0.0416	0.0447	1.664	0.1032	0.047	0.0487	1.157	0.2532
Lv2 Language Motor_Left-L_between_centrality_b	16.0497	13.1656	-1.736	0.0893	14.412	11.3126	-2.397	0.0207	10.6418	8.5983	-2.266	0.0283
Lv2 Language Motor_Left-G_between_centrality_b	277.6573	258.2449	-0.868	0.39	204.6872	193.6583	-0.671	0.5055	151.0896	149.3745	-0.203	0.8397
Lv2 Language Motor_Left-assortativity_b	0.2323	0.2923	1.855	0.0701	0.2381	0.3144	2.01	0.0505	0.1735	0.3239	3.69	0.0006
Lv2 Language Motor_Left-participation_coef	0.6557	0.679	1.452	0.1535	0.7287	0.7422	1.095	0.2795	0.7743	0.7753	0.184	0.8551
Lv2 Language Motor_Right-L_density	0.49	0.5411	2.572	0.0135	0.6002	0.6253	1.149	0.2567	0.6971	0.7	0.143	0.8872
Lv2 Language Motor_Right-G_density	0.1885	0.1868	-0.168	0.8674	0.294	0.2746	-1.645	0.107	0.4016	0.3647	-2.898	0.0058
Lv2 Language Motor_Right-L_global_efficiency	0.7092	0.7245	0.931	0.3567	0.7871	0.788	0.061	0.9514	0.8443	0.8375	-0.586	0.5609
Lv2 Language Motor_Right-L_nodal_efficiency	0.803	0.8386	2.626	0.0118	0.8593	0.8698	1.148	0.2572	0.9003	0.9078	1	0.3228
Lv2 Language Motor_Right-G_nodal_efficiency	0.7565	0.7892	3.383	0.0015	0.8016	0.8156	2.622	0.0119	0.8273	0.8362	1.999	0.0516
Lv2 Language Motor_Right-L_shortest_path_b	1.6655	1.5827	-1.702	0.0956	1.4808	1.4638	-0.362	0.719	1.3299	1.3363	0.208	0.8361
Lv2 Language Motor_Right-G_shortest_path_b	2.1006	2.1446	0.626	0.5346	1.7869	1.8481	1.811	0.0769	1.6156	1.6732	2.81	0.0073
Lv2 Language Motor_Right-L_clustering_coef_b	0.7022	0.7605	4.319	0.0001	0.7686	0.799	2.705	0.0096	0.8225	0.8429	1.927	0.0603
Lv2 Language Motor_Right-G_clustering_coef_b	0.5656	0.6121	4.096	0.0002	0.6174	0.6429	2.647	0.0111	0.6573	0.6754	2.02	0.0494
Lv2 Language Motor_Right-L_eigen_centrality_b	0.1977	0.1966	-0.634	0.5294	0.2046	0.2025	-1.362	0.1799	0.2096	0.2069	-2.269	0.0281
Lv2 Language Motor_Right-G_eigen_centrality_b	0.0363	0.0405	1.496	0.1417	0.0437	0.0452	0.651	0.5181	0.049	0.0485	-0.242	0.8096
Lv2 Language Motor_Right-L_between_centrality_b	12.7453	11.2298	-1.821	0.0753	9.5921	9.1863	-0.432	0.6682	6.5983	6.6936	0.156	0.8768
Lv2 Language Motor_Right-G_between_centrality_b	253.7508	254.5844	0.068	0.9459	194.3149	187.9972	-0.64	0.5254	157.2438	141.9401	-2.529	0.015
Lv2 Language Motor_Right-assortativity_b	0.0893	0.1464	2.09	0.0423	0.0275	0.1197	3.275	0.002	-0.0278	0.0826	4.434	0.0001
Lv2 Language Motor_Right-participation_coef	0.6784	0.6892	0.699	0.4882	0.7494	0.7441	-0.518	0.6067	0.7804	0.7706	-1.659	0.1041
Lv1 Word Recognition_Left-L_density	0.3332	0.3047	-1.884	0.0661	0.4405	0.4029	-2.018	0.0495	0.5438	0.5045	-2.039	0.0473
Lv1 Word Recognition_Left-G_density	0.2153	0.1829	-4.303	0.0001	0.3228	0.2773	-4.814	<0.0001	0.4295	0.3773	-5.098	<0.0001
Lv1 Word Recognition_Left-L_global_efficiency	0.5878	0.5472	-2.219	0.0316	0.6891	0.6575	-2.155	0.0365	0.7615	0.7362	-2.11	0.0404
Lv1 Word Recognition_Left-L_nodal_efficiency	0.7363	0.7319	-0.242	0.8101	0.8074	0.7988	-0.6	0.5514	0.8534	0.8519	-0.189	0.8512
Lv1 Word Recognition_Left-G_nodal_efficiency	0.753	0.7485	-0.407	0.6859	0.7974	0.7984	0.167	0.8683	0.8295	0.8261	-0.696	0.4898
Lv1 Word Recognition_Left-L_shortest_path_b	2.0007	2.155	2.17	0.0353	1.7306	1.8587	2.349	0.0233	1.5156	1.5869	2.065	0.0447
Lv1 Word Recognition_Left-G_shortest_path_b	2.0216	2.1548	3.501	0.0011	1.7544	1.8508	3.732	0.0005	1.5896	1.661	4.4	0.0001
Lv1 Word Recognition_Left-L_clustering_coef_b	0.6233	0.6316	0.5	0.6198	0.6862	0.6853	-0.056	0.9557	0.7298	0.7329	0.247	0.8062
Lv1 Word Recognition_Left-G_clustering_coef_b	0.5733	0.5747	0.126	0.9005	0.6225	0.6226	0.014	0.9892	0.6647	0.6592	-0.543	0.59
Lv1 Word Recognition_Left-L_eigen_centrality_b	0.1563	0.1519	-2.003	0.0512	0.1679	0.1631	-2.513	0.0156	0.1756	0.1727	-2.043	0.0469
Lv1 Word Recognition_Left-G_eigen_centrality_b	0.0436	0.0396	-2.011	0.0503	0.0498	0.0457	-2.85	0.0066	0.054	0.0504	-3.223	0.0024
Lv1 Word Recognition_Left-L_between_centrality_b	25.4379	28.295	1.421	0.1621	19.4658	22.7516	2.342	0.0236	13.9084	15.7981	2.03	0.0482
Lv1 Word Recognition_Left-G_between_centrality_b	244.13	263.0589	1.851	0.0708	194.4253	198.7665	0.572	0.57	159.0917	162.7229	0.658	0.5138
Lv1 Word Recognition_Left-assortativity_b	0.2924	0.3579	1.89	0.0652	0.2077	0.3018	3.038	0.004	0.1452	0.1947	1.721	0.0922
Lv1 Word Recognition_Left-participation_coef	0.7368	0.7053	-2.435	0.0189	0.7942	0.7773	-1.839	0.0725	0.8296	0.8144	-2.827	0.007
Lv1 Word Recognition_Right-L_density	0.322	0.289	-1.972	0.0548	0.4336	0.3913	-2.421	0.0196	0.5416	0.4917	-2.841	0.0067
Lv1 Word Recognition_Right-G_density	0.2164	0.187	-3.465	0.0012	0.3239	0.2829	-4.251	0.0001	0.4311	0.3825	-4.842	<0.0001
Lv1 Word Recognition_Right-L_global_efficiency	0.5784	0.5397	-2.19	0.0337	0.6845	0.6525	-2.462	0.0177	0.7584	0.7275	-2.814	0.0072
Lv1 Word Recognition_Right-L_nodal_efficiency	0.7198	0.7304	0.697	0.4893	0.8055	0.7947	-0.96	0.3423	0.8546	0.8445	-1.345	0.1853
Lv1 Word Recognition_Right-G_nodal_efficiency	0.744	0.7567	1.553	0.1274	0.7997	0.8003	0.085	0.9325	0.8312	0.8277	-0.834	0.4089
Lv1 Word Recognition_Right-L_shortest_path_b	2.0458	2.1917	2.084	0.0429	1.7717	1.8469	1.517	0.1362	1.5363	1.6024	1.788	0.0804
Lv1 Word Recognition_Right-G_shortest_path_b	2.0532	2.1656	2.671	0.0105	1.7664	1.8484	3.03	0.004	1.5905	1.6559	4.13	0.0002
Lv1 Word Recognition_Right-L_clustering_coef_b	0.6115	0.6251	1.026	0.3105	0.6847	0.6718	-1.094	0.2797	0.7377	0.7214	-1.515	0.1369
Lv1 Word Recognition_Right-G_clustering_coef_b	0.5717	0.584	1.262	0.2135	0.6261	0.6228	-0.339	0.7361	0.6681	0.6604	-0.926	0.3594
Lv1 Word Recognition_Right-L_eigen_centrality_b	0.1529	0.1481	-2.015	0.0499	0.1644	0.162	-1.305	0.1986	0.1723	0.1702	-1.531	0.1327
Lv1 Word Recognition_Right-G_eigen_centrality_b	0.0454	0.0414	-1.845	0.0716	0.0506	0.0472	-2.065	0.0447	0.0544	0.0514	-2.331	0.0243
Lv1 Word Recognition_Right-L_between_centrality_b	27.3598	30.8006	1.712	0.0939	21.3403	23.2669	1.436	0.158	14.9985	16.7466	1.691	0.0977
Lv1 Word Recognition_Right-G_between_centrality_b	267.7934	273.2285	0.47	0.6408	199.3061	204.951	0.806	0.4247	158.1928	164.7491	1.281	0.2069
Lv1 Word Recognition_Right-assortativity_b	0.2855	0.3108	0.748	0.4585	0.2022	0.2272	0.792	0.4325	0.1315	0.1597	1.019	0.3136
Lv1 Word Recognition_Right-participation_coef	0.7304	0.7134	-1.396	0.1697	0.797	0.7776	-2.277	0.0276	0.8313	0.8147	-3.178	0.0027
Lv1 Semantic Processing_Left-L_density	0.397	0.4288	1.528	0.1334	0.5195	0.5518	1.555	0.1269	0.6255	0.656	1.477	0.1467
Lv1 Semantic Processing_Left-G_density	0.2066	0.188	-2.166	0.0356	0.3146	0.2836	-3.043	0.0039	0.4208	0.3837	-3.387	0.0015
Lv1 Semantic Processing_Left-L_global_efficiency	0.6543	0.664	0.512	0.6114	0.742	0.7601	1.271	0.2103	0.8069	0.8228	1.37	0.1776
Lv1 Semantic Processing_Left-L_nodal_efficiency	0.7821	0.7953	0.669	0.5071	0.8412	0.8549	1.165	0.2503	0.8815	0.895	1.82	0.0754
Lv1 Semantic Processing_Left-G_nodal_efficiency	0.7558	0.7668	0.805	0.4248	0.7977	0.8014	0.444	0.6588	0.8263	0.8251	-0.26	0.7962
Lv1 Semantic Processing_Left-L_shortest_path_b	1.8134	1.7616	-0.902	0.3717	1.5595	1.5226	-1.02	0.313	1.4039	1.3761	-0.946	0.3491
Lv1 Semantic Processing_Left-G_shortest_path_b	2.0313	2.1279	2.082	0.0431	1.7712	1.8227	1.355	0.1821	1.5992	1.6453	2.394	0.0209
Lv1 Semantic Processing_Left-L_clustering_coef_b	0.6546	0.6889	1.884	0.066	0.7213	0.7473	1.843	0.0719	0.7736	0.8022	2.478	0.017
Lv1 Semantic Processing_Left-G_clustering_coef_b	0.5662	0.583	1.381	0.1741	0.615	0.6183	0.34	0.7351	0.6572	0.6544	-0.326	0.7458
Lv1 Semantic Processing_Left-L_eigen_centrality_b	0.1847	0.1844	-0.127	0.8994	0.1935	0.1935	-0.034	0.9732	0.1992	0.1992	-0.033	0.974
Lv1 Semantic Processing_Left-G_eigen_centrality_b	0.0413	0.0423	0.446	0.6575	0.0481	0.0474	-0.355	0.7241	0.0524	0.0517	-0.474	0.6376
Lv1 Semantic Processing_Left-L_between_centrality_b	16.8147	16.0529	-0.748	0.4585	12.0756	11.3762	-0.951	0.3465	8.8677	8.2741	-0.918	0.3636
Lv1 Semantic Processing_Left-G_between_centrality_b	295.9858	294.3796	-0.109	0.9137	219.8769	221.4146	0.129	0.8977	173.076	174.408	0.177	0.8606
Lv1 Semantic Processing_Left-assortativity_b	0.0745	0.1582	2.581	0.0132	0.0344	0.0939	1.893	0.0647	-0.0067	0.0208	1.043	0.3027
Lv1 Semantic Processing_Left-participation_coef	0.7741	0.7539	-1.189	0.2408	0.8206	0.8117	-0.721	0.4747	0.8488	0.841	-1.328	0.1909
Lv1 Semantic Processing_Right-L_density	0.3626	0.3544	-0.517	0.6074	0.481	0.471	-0.567	0.5738	0.5856	0.5746	-0.626	0.5347
Lv1 Semantic Processing_Right-G_density	0.2021	0.1801	-2.444	0.0185	0.3042	0.2724	-3.056	0.0038	0.4058	0.3692	-3.36	0.0016
Lv1 Semantic Processing_Right-L_global_efficiency	0.6403	0.6277	-0.836	0.4078	0.7246	0.7158	-0.737	0.4649	0.7867	0.7793	-0.732	0.4679
Lv1 Semantic Processing_Right-L_nodal_efficiency	0.7864	0.7916	0.391	0.6977	0.8388	0.8424	0.444	0.6595	0.8733	0.8721	-0.202	0.8405
Lv1 Semantic Processing_Right-G_nodal_efficiency	0.7667	0.7723	0.688	0.4948	0.8073	0.8062	-0.238	0.8129	0.8311	0.8264	-1.187	0.2413
Lv1 Semantic Processing_Right-L_shortest_path_b	1.8748	1.8666	-0.127	0.8995	1.6094	1.6203	0.304	0.7624	1.4425	1.4695	1.034	0.3067
Lv1 Semantic Processing_Right-G_shortest_path_b	2.0453	2.1359	1.842	0.0721	1.7718	1.8352	2.131	0.0386	1.6124	1.6655	2.878	0.0061
Lv1 Semantic Processing_Right-L_clustering_coef_b	0.6515	0.6578	0.47	0.6403	0.707	0.7149	0.72	0.4755	0.7565	0.7579	0.133	0.8946

Lv1 Semantic Processing_Right-G_clustering_coef_b	0.5809	0.5865	0.564	0.5758	0.6271	0.6247	-0.278	0.7823	0.6653	0.6586	-0.882	0.3823
Lv1 Semantic Processing_Right-L_eigen_centrality_b	0.1525	0.1503	-0.938	0.3535	0.1609	0.1584	-1.889	0.0653	0.1658	0.1637	-2.839	0.0068
Lv1 Semantic Processing_Right-G_eigen_centrality_b	0.0395	0.0388	-0.264	0.7928	0.0455	0.0443	-0.637	0.5272	0.0496	0.0488	-0.534	0.5959
Lv1 Semantic Processing_Right-L_between_centrality_	27.1568	26.7589	-0.24	0.8115	19.3808	19.5692	0.17	0.866	14.1199	14.9644	1.01	0.3181
Lv1 Semantic Processing_Right-G_between_centrality_	262.5144	293.2295	2.237	0.0303	197.4763	210.9074	1.569	0.1237	156.1224	163.731	1.326	0.1914
Lv1 Semantic Processing_Right-assortativity_b	0.1123	0.2008	2.683	0.0102	0.0659	0.1482	2.903	0.0057	0.0232	0.1067	3.572	0.0009
Lv1 Semantic Processing_Right-participation_coef	0.753	0.7338	-1.445	0.1553	0.8125	0.7958	-2.015	0.0499	0.8413	0.8287	-2.428	0.0193
Lv1 Syntatic Processing_Left-L_density	0.4907	0.5317	2.127	0.0389	0.5907	0.6472	2.95	0.005	0.6675	0.7376	3.734	0.0005
Lv1 Syntatic Processing_Left-G_density	0.2694	0.2471	-2.706	0.0096	0.3695	0.3459	-2.637	0.0114	0.4612	0.4404	-2.28	0.0274
Lv1 Syntatic Processing_Left-L_global_efficiency	0.7346	0.7571	2.126	0.0391	0.7919	0.8218	2.981	0.0046	0.8326	0.8684	3.736	0.0005
Lv1 Syntatic Processing_Left-L_nodal_efficiency	0.8658	0.8774	2.204	0.0327	0.8861	0.9023	2.99	0.0045	0.9039	0.9222	3.242	0.0022
Lv1 Syntatic Processing_Left-G_nodal_efficiency	0.8137	0.8092	-0.896	0.3748	0.832	0.8228	-1.858	0.0697	0.8495	0.8383	-2.354	0.023
Lv1 Syntatic Processing_Left-L_shortest_path_b	1.5711	1.5187	-1.975	0.0544	1.4299	1.3638	-2.983	0.0046	1.3396	1.265	-3.719	0.0006
Lv1 Syntatic Processing_Left-G_shortest_path_b	1.895	1.9667	3.069	0.0036	1.6807	1.7233	2.873	0.0062	1.55	1.5769	2.635	0.0115
Lv1 Syntatic Processing_Left-L_clustering_coef_b	0.7349	0.7602	2.432	0.0191	0.7735	0.8049	2.914	0.0055	0.8084	0.8446	3.24	0.0023
Lv1 Syntatic Processing_Left-G_clustering_coef_b	0.63	0.6218	-0.794	0.4314	0.6645	0.6463	-1.83	0.0739	0.6992	0.6768	-2.346	0.0235
Lv1 Syntatic Processing_Left-L_eigen_centrality_b	0.1312	0.1306	-0.815	0.4191	0.1343	0.1346	0.543	0.59	0.1359	0.1366	1.799	0.0787
Lv1 Syntatic Processing_Left-G_eigen_centrality_b	0.0581	0.0559	-0.767	0.4468	0.059	0.059	0.03	0.9766	0.0592	0.0606	0.958	0.3432
Lv1 Syntatic Processing_Left-L_between_centrality_b	28.5516	25.9037	-2.001	0.0515	21.4936	18.1876	-2.983	0.0046	16.9821	13.2523	-3.719	0.0006
Lv1 Syntatic Processing_Left-G_between_centrality_b	226.993	279.2623	5.431	<0.0001	172.5033	214.8439	5.527	<0.0001	138.7505	171.3831	5.162	<0.0001
Lv1 Syntatic Processing_Left-assortativity_b	0.1609	0.1411	-0.941	0.3518	0.1063	0.0724	-1.79	0.0802	0.0668	0.0146	-2.786	0.0078
Lv1 Syntatic Processing_Left-participation_coef	0.7451	0.692	-6.252	<0.0001	0.7885	0.7498	-5.926	<0.0001	0.8149	0.7855	-5.762	<0.0001
Lv1 Syntatic Processing_Right-L_density	0.531	0.5201	-0.627	0.5339	0.6307	0.6352	0.269	0.7892	0.7012	0.7231	1.396	0.1696
Lv1 Syntatic Processing_Right-G_density	0.2982	0.2587	-5.208	<0.0001	0.4021	0.3608	-5.257	<0.0001	0.493	0.4574	-4.607	<0.0001
Lv1 Syntatic Processing_Right-L_global_efficiency	0.7559	0.7515	-0.475	0.6369	0.8124	0.8157	0.385	0.7018	0.8496	0.8611	1.45	0.1541
Lv1 Syntatic Processing_Right-L_nodal_efficiency	0.8758	0.8738	-0.383	0.7037	0.8989	0.8977	-0.219	0.8274	0.9155	0.918	0.529	0.5996
Lv1 Syntatic Processing_Right-G_nodal_efficiency	0.8109	0.8005	-2.096	0.0418	0.8314	0.8166	-2.901	0.0057	0.8503	0.8335	-3.487	0.0011
Lv1 Syntatic Processing_Right-L_shortest_path_b	1.5267	1.5286	0.085	0.9328	1.3873	1.3764	-0.59	0.5581	1.3049	1.2796	-1.549	0.1284
Lv1 Syntatic Processing_Right-G_shortest_path_b	1.8502	1.936	4.451	0.0001	1.6416	1.7002	4.809	<0.0001	1.5164	1.557	4.777	<0.0001
Lv1 Syntatic Processing_Right-L_clustering_coef_b	0.755	0.7516	-0.323	0.7482	0.7987	0.7965	-0.22	0.827	0.8312	0.8367	0.575	0.5684
Lv1 Syntatic Processing_Right-G_clustering_coef_b	0.6239	0.6041	-1.944	0.0581	0.6631	0.6338	-2.872	0.0062	0.7007	0.6672	-3.475	0.0011
Lv1 Syntatic Processing_Right-L_eigen_centrality_b	0.133	0.1321	-1.166	0.2497	0.1357	0.136	0.558	0.5796	0.1373	0.138	1.678	0.1002
Lv1 Syntatic Processing_Right-G_eigen_centrality_b	0.0667	0.06	-3.61	0.0008	0.0655	0.0628	-1.889	0.0654	0.0641	0.0637	-0.334	0.7398
Lv1 Syntatic Processing_Right-L_between_centrality_b	25.8104	25.8765	0.063	0.9497	18.9774	18.4443	-0.59	0.5581	14.9383	13.7	-1.549	0.1284
Lv1 Syntatic Processing_Right-G_between_centrality_b	266.6644	321.3793	3.786	0.0005	196.4572	240.2334	4.132	0.0002	152.3936	188.4262	4.718	<0.0001
Lv1 Syntatic Processing_Right-assortativity_b	0.1338	0.1476	0.569	0.5723	0.1076	0.075	-1.462	0.1507	0.0727	0.0173	-2.623	0.0118
Lv1 Syntatic Processing_Right-participation_coef	0.7663	0.7147	-8.799	<0.0001	0.8035	0.7669	-8.338	<0.0001	0.8252	0.799	-7.48	<0.0001
Lv1 Acoustic Processing_Left-L_density	0.8039	0.8338	1.317	0.1943	0.8816	0.8899	0.48	0.6338	0.9242	0.9314	0.585	0.5618
Lv1 Acoustic Processing_Left-G_density	0.2586	0.2458	-1.236	0.2229	0.3776	0.344	-2.963	0.0049	0.4885	0.4425	-3.945	0.0003
Lv1 Acoustic Processing_Left-L_global_efficiency	0.8902	0.9121	1.562	0.1253	0.9405	0.9443	0.436	0.6647	0.962	0.9656	0.581	0.564
Lv1 Acoustic Processing_Left-L_nodal_efficiency	0.9181	0.94	1.724	0.0915	0.9592	0.9607	0.234	0.8157	0.9758	0.9762	0.11	0.9129
Lv1 Acoustic Processing_Left-G_nodal_efficiency	0.8113	0.8182	0.838	0.4064	0.8368	0.8362	-0.111	0.9119	0.8487	0.8493	0.147	0.8839
Lv1 Acoustic Processing_Left-L_shortest_path_b	1.2063	1.1778	-0.987	0.3287	1.1203	1.114	-0.351	0.727	1.0763	1.0691	-0.574	0.5688
Lv1 Acoustic Processing_Left-G_shortest_path_b	1.93	1.9818	1.299	0.2006	1.6776	1.7278	2.157	0.0364	1.5218	1.5752	3.761	0.0005
Lv1 Acoustic Processing_Left-L_clustering_coef_b	0.8676	0.8985	1.942	0.0584	0.9218	0.9277	0.518	0.6072	0.9516	0.9525	0.12	0.9048
Lv1 Acoustic Processing_Left-G_clustering_coef_b	0.6431	0.6489	0.5	0.6193	0.6746	0.6747	0.006	0.9951	0.6977	0.6988	0.12	0.9052
Lv1 Acoustic Processing_Left-L_eigen_centrality_b	0.3084	0.3107	1.534	0.132	0.3128	0.3132	0.517	0.6077	0.3143	0.3146	0.638	0.527
Lv1 Acoustic Processing_Left-G_eigen_centrality_b	0.0599	0.0623	0.792	0.4328	0.0635	0.063	-0.198	0.8437	0.065	0.0636	-0.759	0.4515
Lv1 Acoustic Processing_Left-L_between_centrality_b	1.7783	1.6	-0.715	0.4783	1.0826	1.0261	-0.351	0.727	0.687	0.6217	-0.574	0.5688
Lv1 Acoustic Processing_Left-G_between_centrality_b	229.2091	219.6771	-0.409	0.6847	186.1814	171.6963	-1.271	0.2104	158.037	143.5346	-1.664	0.103
Lv1 Acoustic Processing_Left-participation_coef	0.8158	0.8215	0.459	0.6482	0.8418	0.8439	0.316	0.7532	0.8543	0.8558	0.393	0.6959
Lv1 Acoustic Processing_Right-L_density	0.9326	0.9283	-0.224	0.8237	0.9522	0.9543	0.129	0.8981	0.9783	0.9717	-0.553	0.5831
Lv1 Acoustic Processing_Right-G_density	0.3189	0.2857	-2.541	0.0146	0.4353	0.3898	-3.38	0.0015	0.5337	0.4889	-3.321	0.0018
Lv1 Acoustic Processing_Right-L_global_efficiency	0.9645	0.9569	-0.518	0.6072	0.975	0.9772	0.248	0.805	0.9891	0.9859	-0.553	0.5831
Lv1 Acoustic Processing_Right-L_nodal_efficiency	0.9492	0.9481	-0.062	0.9511	0.9692	0.9587	-0.523	0.6037	0.9873	0.9902	0.443	0.6597
Lv1 Acoustic Processing_Right-G_nodal_efficiency	0.7987	0.8034	0.707	0.4832	0.8197	0.8188	-0.157	0.8756	0.8391	0.8357	-0.694	0.4912
Lv1 Acoustic Processing_Right-L_shortest_path_b	1.0783	1.063	-0.805	0.4249	1.0543	1.0457	-0.455	0.6514	1.0217	1.0283	0.553	0.5831
Lv1 Acoustic Processing_Right-G_shortest_path_b	1.8279	1.888	1.709	0.0943	1.6036	1.6631	2.513	0.0156	1.4729	1.5246	3.276	0.002
Lv1 Acoustic Processing_Right-L_clustering_coef_b	0.9319	0.9333	0.082	0.9352	0.9572	0.9514	-0.285	0.7766	0.9812	0.9804	-0.081	0.9358
Lv1 Acoustic Processing_Right-G_clustering_coef_b	0.6083	0.6114	0.239	0.8124	0.6405	0.6384	-0.183	0.856	0.6786	0.6716	-0.719	0.4757
Lv1 Acoustic Processing_Right-L_eigen_centrality_b	0.4446	0.4428	-0.759	0.4516	0.4454	0.4454	0.101	0.9198	0.4464	0.4464	-0.074	0.9412
Lv1 Acoustic Processing_Right-G_eigen_centrality_b	0.0756	0.0725	-0.75	0.4571	0.0731	0.0712	-0.667	0.5083	0.0703	0.07	-0.147	0.8841
Lv1 Acoustic Processing_Right-L_between_centrality_b	0.313	0.2522	-0.805	0.4249	0.2174	0.1826	-0.455	0.6514	0.087	0.113	0.553	0.5831
Lv1 Acoustic Processing_Right-G_between_centrality_l	430.331	405.2303	-0.316	0.7531	316.1713	285.7298	-0.707	0.4833	225.8241	212.6327	-0.632	0.5308
Lv1 Acoustic Processing_Right-participation_coef	0.8385	0.8356	-0.49	0.6263	0.8506	0.8494	-0.284	0.7778	0.859	0.8574	-0.522	0.6043
Lv1 Motoric Language_Left-L_density	0.3917	0.4449	3.169	0.0027	0.4986	0.5331	1.907	0.0629	0.591	0.6064	0.931	0.357
Lv1 Motoric Language_Left-G_density	0.1714	0.177	0.631	0.5313	0.2735	0.2671	-0.631	0.5313	0.378	0.3609	-1.61	0.1144
Lv1 Motoric Language_Left-L_global_efficiency	0.596	0.6168	1.143	0.2591	0.6983	0.6997	0.082	0.9347	0.7775	0.7546	-1.765	0.0844
Lv1 Motoric Language_Left-L_nodal_efficiency	0.7307	0.7644	2.059	0.0453	0.8085	0.8308	1.625	0.1112	0.8564	0.8759	2.157	0.0364
Lv1 Motoric Language_Left-G_nodal_efficiency	0.7099	0.7456	2.822	0.0071	0.7713	0.7971	2.373	0.022	0.8181	0.8261	1.182	0.2434
Lv1 Motoric Language_Left-L_shortest_path_b	1.8785	1.7189	-2.119	0.0396	1.7548	1.5877	-2.484	0.0168	1.5321	1.4422	-2.055	0.0457
Lv1 Motoric Language_Left-G_shortest_path_b	2.1319	2.181	1.258	0.215	1.8607	1.8949	0.878	0.3848	1.6578	1.6955	1.846	0.0715
Lv1 Motoric Language_Left-L_clustering_coef_b	0.644	0.6932	2.87	0.0062	0.7232	0.7596	2.801	0.0075	0.7743	0.8098	3.06	0.0037
Lv1 Motoric Language_Left-G_clustering_coef_b	0.536	0.5803	3.209	0.0025	0.597	0.6257	2.226	0.0311	0.6544	0.6643	0.899	0.3734
Lv1 Motoric Language_Left-L_eigen_centrality_b	0.1856	0.1884	1.502	0.1401	0.194	0.1955	0.814	0.4197	0.2013	0.2006	-0.518	0.6067
Lv1 Motoric Language_Left-G_eigen_centrality_b	0.0342	0.0395	2.321	0.0249	0.0416	0.0447	1.664	0.1032	0.047	0.0487	1.157	0.2532
Lv1 Motoric Language_Left-L_between_centrality_b	16.0497	13.1656	-1.736	0.0893	14.412	11.3126	-2.397	0.0207	10.6418	8.5983	-2.266	0.0283
Lv1 Motoric Language_Left-G_between_centrality_b	277.6573	258.2449	-0.868	0.39	204.6872	193.6583	-0.671	0.5055	151.0896	149.3745	-0.203	0.8397
Lv1 Motoric Language_Left-assortativity_b	0.2323	0.2923	1.855	0.0701	0.2381	0.3144	2.01	0.0505	0.1735	0.3239	3.69	0.0006
Lv1 Motoric Language_Left-participation_coef	0.693	0.7198	1.58	0.1211	0.7771	0.7929	1.195	0.2384	0.8293	0.832	0.438	0.6636
Lv1 Motoric Language_Right-L_density	0.49	0.5411	2.572	0.0135	0.6002	0.6253	1.149	0.2567	0.6971	0.7	0.143	0.8872
Lv1 Motoric Language_Right-G_density	0.1885	0.1868	-0.168	0.8674	0.294	0.2746	-1.645	0.107	0.4016	0.3647	-2.898	0.0058
Lv1 Motoric Language_Right-L_global_efficiency	0.7092	0.7245	0.931	0.3567	0.7871	0.788	0.061	0.9514	0.8443	0.8375	-0.586	0.5609
Lv1 Motoric Language_Right-L_nodal_efficiency	0.803	0.8386	2.626	0.0118	0.8593	0.8698	1.148	0.2572	0.9003	0.9078	1	0.3228
Lv1 Motoric Language_Right-G_nodal_efficiency	0.7565	0.7892	3.383	0.0015	0.8016	0.8156	2.622	0.0119	0.8273	0.8362	1.999	0.0516
Lv1 Motoric Language_Right-L_shortest_path_b	1.6655	1.5827	-1.702	0.0956	1.4808	1.4638	-0.362	0.719	1.3299	1.3363	0.208	0.8361
Lv1 Motoric Language_Right-G_shortest_path_b	2.1006	2.1446	0.626	0.5346	1.7869	1.8481	1.811	0.0769	1.6156	1.6732	2.81	0.0073
Lv1 Motoric Language_Right-L_clustering_coef_b	0.7022	0.7605	4.319	0.0001	0.7686	0.799	2.705	0.0096	0.8225	0.8429	1.927	0.0603
Lv1 Motoric Language_Right-G_clustering_coef_b	0.5656	0.6121	4.096	0.0002	0.6174	0.6429	2.647	0.0111	0.6573	0.6754	2.02	0.0494
Lv1 Motoric Language_Right-L_eigen_centrality_b	0.1977	0.1966	-0.634	0.5294	0.2046	0.2025	-1.362	0.1799	0.2096	0.2069	-2.269	0.0281
Lv1 Motoric Language_Right-G_eigen_centrality_b	0.0363	0.0405	1.496	0.1417	0.0437	0.0452	0.651	0.5181	0.049	0.0485	-0.242	0.8096
Lv1 Motoric Language_Right-L_between_centrality_b	12.7453	11.2298	-1.821	0.0753	9.5921	9.1863	-0.432	0.6682	6.5983	6.6936	0.156	0.8768
Lv1 Motoric Language_Right-G_between_centrality_b	253.7508	254.5844	0.068	0.9459	194.3149	187.9972	-0.64	0.5254	157.2438	141.9401	-2.529	0.015
Lv1 Motoric Language_Right-assortativity_b	0.0893	0.1464	2.09	0.0423	0.0275	0.1197	3.275	0.002	-0.0278	0.0826	4.434	0.0001
Lv1 Motoric Language_Right-participation_coef	0.7233	0.7381	0.925	0.3597	0.8042	0.8012	-0.29	0.7734	0.8413	0.833	-1.689	0.0982

Supplementary Table 5

Linear mixed effect modelling for binary graph metrics adjusted by global functional connectivity (no global signal scaling)

		Word recognition WC	Word recognition WG	Motoric language WC	Motoric language WG	T-value Module	p-value Module	T-value Task	p-value Task	T-value Module x Task	p-value Module x Task
20 % threshold											
	L_density	0.278	0.226	0.356	0.380	0.167	0.8672	-2.883	0.0044	2.867	0.0046
	G_density	1.835	1.440	1.666	1.528	-5.968	< 0.0001	-4.734	< 0.0001	1.940	0.0539
	L_global_efficiency	0.490	0.416	0.557	0.546	-1.958	0.0518	-3.617	0.0004	2.162	0.0319
	L_nodal_efficiency	0.658	0.621	0.693	0.734	-2.049	0.0419	-2.292	0.0231	1.288	0.1994
	G_nodal_efficiency	0.706	0.685	0.678	0.721	-3.456	0.0007	-1.955	0.0522	0.721	0.4721
	L_shortest_path	2.159	2.248	1.896	1.765	-2.087	0.0383	1.866	0.0637	-2.548	0.0117
	G_shortest_path	2.191	2.406	2.343	2.433	2.747	0.0066	4.089	0.0001	-1.431	0.1543
	L_clustering_coef	0.570	0.543	0.607	0.664	-1.482	0.1400	-1.673	0.0961	1.422	0.1568
	G_clustering_coef	0.566	0.549	0.532	0.589	-2.955	0.0036	-1.640	0.1027	0.766	0.4447
	L_eigen_centrality	0.146	0.137	0.174	0.173	10.613	< 0.0001	-3.018	0.0029	1.751	0.0817
	G_eigen_centrality	0.042	0.036	0.027	0.030	-4.651	< 0.0001	-2.303	0.0224	1.382	0.1685
	L_between_centrality	26.828	27.129	16.427	13.588	-5.769	< 0.0001	0.048	0.9617	-1.043	0.2984
	G_between_centrality	289.211	336.108	280.883	260.204	1.696	0.0917	1.805	0.0727	-1.978	0.0495
	assortativity	0.269	0.359	0.186	0.241	-2.654	0.0087	1.168	0.2443	0.221	0.8252
	participation_coef	0.657	0.605	0.587	0.590	-5.464	< 0.0001	-3.938	0.0001	2.270	0.0244
30 % threshold											
	L_density	0.379	0.312	0.451	0.462	-0.135	0.8930	-3.016	0.0029	2.401	0.0174
	G_density	2.794	2.253	2.728	2.395	-5.775	< 0.0001	-5.016	< 0.0001	1.592	0.1132
	L_global_efficiency	0.618	0.542	0.652	0.631	-2.179	0.0306	-3.207	0.0016	1.316	0.1899
	L_nodal_efficiency	0.746	0.725	0.769	0.785	-2.209	0.0285	-1.361	0.1752	0.755	0.4511
	G_nodal_efficiency	0.780	0.768	0.760	0.782	-3.192	0.0017	-1.094	0.2753	0.505	0.6145
	L_shortest_path	1.888	2.050	1.740	1.654	-0.674	0.5013	3.132	0.0020	-3.148	0.0019
	G_shortest_path	1.888	2.056	1.979	2.133	3.619	0.0004	4.703	< 0.0001	-0.820	0.4134
	L_clustering_coef	0.648	0.629	0.679	0.716	-0.824	0.4108	-0.914	0.3621	0.849	0.3968
	G_clustering_coef	0.640	0.626	0.610	0.650	-2.566	0.0111	-1.089	0.2775	0.813	0.4172
	L_eigen_centrality	0.157	0.150	0.183	0.180	11.821	< 0.0001	-3.115	0.0021	1.529	0.1280
	G_eigen_centrality	0.047	0.041	0.034	0.035	-4.805	< 0.0001	-2.476	0.0142	1.233	0.2192
	L_between_centrality	23.165	25.981	15.026	12.940	-4.268	< 0.0001	2.890	0.0043	-3.059	0.0026
	G_between_centrality	236.220	256.876	209.345	215.594	0.824	0.4109	1.685	0.0937	-1.145	0.2538
	assortativity	0.224	0.294	0.148	0.259	-0.926	0.3555	1.488	0.1384	0.319	0.7498
	participation_coef	0.739	0.687	0.699	0.692	-3.737	0.0003	-3.882	0.0001	1.771	0.0782
40 % threshold											
	L_density	0.479	0.405	0.541	0.539	-0.395	0.6930	-3.264	0.0013	2.151	0.0328
	G_density	3.760	3.126	3.912	3.363	-5.536	< 0.0001	-5.122	< 0.0001	1.273	0.2045
	L_global_efficiency	0.710	0.645	0.727	0.702	-1.860	0.0646	-3.143	0.0020	0.765	0.4455
	L_nodal_efficiency	0.819	0.788	0.828	0.842	-2.425	0.0163	-1.369	0.1726	0.255	0.7988
	G_nodal_efficiency	0.831	0.818	0.820	0.834	-2.966	0.0034	-2.113	0.0360	1.327	0.1862
	L_shortest_path	1.668	1.831	1.559	1.567	1.567	0.1189	2.907	0.0041	-3.414	0.0008
	G_shortest_path	1.684	1.829	1.750	1.888	4.494	< 0.0001	5.108	< 0.0001	-1.169	0.2438
	L_clustering_coef	0.718	0.688	0.742	0.776	-0.808	0.4202	-1.243	0.2156	0.706	0.4813
	G_clustering_coef	0.695	0.679	0.677	0.704	-2.016	0.0453	-2.407	0.0171	1.229	0.2208
	L_eigen_centrality	0.166	0.160	0.190	0.186	14.936	< 0.0001	-3.010	0.0030	1.510	0.1329
	G_eigen_centrality	0.051	0.046	0.040	0.039	-4.964	< 0.0001	-2.675	0.0082	1.098	0.2738
	L_between_centrality	18.436	22.250	11.847	11.854	-1.844	0.0668	3.055	0.0026	-3.502	0.0006
	G_between_centrality	184.607	211.582	163.537	167.201	0.304	0.7612	0.730	0.4665	-0.716	0.4752
	assortativity	0.155	0.223	0.143	0.282	0.355	0.7232	2.293	0.0230	-0.200	0.8418
	participation_coef	0.790	0.750	0.778	0.758	-3.197	0.0016	-3.810	0.0002	2.044	0.0424

Supplementary Table 6

Linear mixed effect modelling for binary graph metrics adjusted by global functional connectivity (incl. global signal scaling)

		Word recognition WC	Word recognition WG	Motoric language WC	Motoric language WG	T-value Module	p-value Module	T-value Task	p-value Task	T-value Module x Task	p-value Module x Task
20 % threshold											
	L_density	0.333	0.305	0.392	0.445	3.336	0.0010	-2.223	0.0275	3.296	0.0012
	G_density	0.215	0.183	0.171	0.177	-4.905	0.0306163	-4.477	< 0.0001	3.008	0.0030
	L_global_efficiency	0.588	0.547	0.596	0.617	0.456	0.6488	-2.450	0.0152	2.408	0.0170
	L_nodal_efficiency	0.736	0.732	0.731	0.764	-0.307	0.7588	-0.856	0.3930	1.471	0.1431
	G_nodal_efficiency	0.753	0.749	0.710	0.746	-3.746	0.0002	-1.033	0.3028	2.469	0.0145
	L_shortest_path	2.001	2.155	1.879	1.719	-1.668	0.0971	2.488	0.0138	-3.032	0.0028
	G_shortest_path	2.022	2.155	2.132	2.181	3.052	0.0026	4.208	< 0.0001	-1.644	0.1019
	L_clustering_coef	0.623	0.632	0.644	0.693	1.120	0.2644	-0.276	0.7830	1.556	0.1214
	G_clustering_coef	0.573	0.575	0.536	0.580	-3.244	0.0014	-0.505	0.6140	2.637	0.0091
	L_eigen_centrality	0.156	0.152	0.186	0.188	13.465	0.0306163	-2.227	0.0272	2.311	0.0220
	G_eigen_centrality	0.044	0.040	0.034	0.040	-3.621	0.0004	-2.306	0.0222	2.536	0.0121
	L_between_centrality	25.438	28.295	16.050	13.166	-5.246	0.0306	1.875	0.0624	-2.268	0.0245
	G_between_centrality	244.130	263.059	277.657	258.245	1.973	0.0500	1.087	0.2784	-1.595	0.1124
	assortativity	0.292	0.358	0.232	0.292	-1.673	0.0961	1.495	0.1366	-0.109	0.9132
	participation_coef	0.737	0.705	0.693	0.720	-3.087	0.0023	-2.706	0.0075	2.907	0.0041
30 % threshold											
	L_density	0.441	0.403	0.499	0.533	3.025	0.0029	-2.346	0.0201	2.664	0.0084
	G_density	0.323	0.277	0.273	0.267	-4.487	< 0.0001	-4.908	< 0.0001	2.516	0.0127
	L_global_efficiency	0.689	0.657	0.698	0.700	0.589	0.5569	-2.118	0.0355	1.503	0.1345
	L_nodal_efficiency	0.807	0.799	0.808	0.831	0.070	0.9444	-1.006	0.3156	1.438	0.1522
	G_nodal_efficiency	0.797	0.798	0.771	0.797	-2.904	0.0042	-0.371	0.7110	1.947	0.0531
	L_shortest_path	1.731	1.859	1.755	1.588	0.389	0.6980	2.326	0.0211	-3.362	0.0009
	G_shortest_path	1.754	1.851	1.861	1.895	3.420	0.0008	3.424	0.0008	-1.415	0.1588
	L_clustering_coef	0.686	0.685	0.723	0.760	2.247	0.0258	-0.684	0.4946	1.599	0.1116
	G_clustering_coef	0.622	0.623	0.597	0.626	-2.301	0.0225	-0.535	0.5931	1.826	0.0696
	L_eigen_centrality	0.168	0.163	0.194	0.195	13.772	< 0.0001	-2.529	0.0123	2.327	0.0211
	G_eigen_centrality	0.050	0.046	0.042	0.045	-3.648	0.0003	-2.536	0.0121	2.265	0.0247
	L_between_centrality	19.466	22.752	14.412	11.313	-3.626	0.0004	2.596	0.0102	-3.239	0.0014
	G_between_centrality	194.425	198.767	204.687	193.658	0.835	0.4048	0.179	0.8579	-0.884	0.3777
	assortativity	0.208	0.302	0.238	0.314	0.810	0.4189	2.150	0.0329	-0.337	0.7368
	participation_coef	0.794	0.777	0.777	0.793	-1.628	0.1053	-1.917	0.0568	2.204	0.0288
40 % threshold											
	L_density	0.544	0.504	0.591	0.606	2.555	0.0115	-2.314	0.0218	2.093	0.0378
	G_density	0.429	0.377	0.378	0.361	-4.350	< 0.0001	-5.076	< 0.0001	2.093	0.0377
	L_global_efficiency	0.762	0.736	0.778	0.755	1.302	0.1946	-2.004	0.0466	0.137	0.8909
	L_nodal_efficiency	0.853	0.852	0.856	0.876	0.287	0.7744	-0.521	0.6031	1.436	0.1529
	G_nodal_efficiency	0.830	0.826	0.818	0.826	-2.083	0.0387	-1.177	0.2408	1.468	0.1440
	L_shortest_path	1.516	1.587	1.532	1.442	0.413	0.6802	1.943	0.0536	-2.861	0.0047
	G_shortest_path	1.590	1.661	1.658	1.695	3.864	0.0002	4.341	< 0.0001	-1.351	0.1783
	L_clustering_coef	0.730	0.733	0.774	0.810	3.105	0.0022	-0.334	0.7387	1.595	0.1125
	G_clustering_coef	0.665	0.659	0.654	0.664	-1.095	0.2750	-1.212	0.2270	1.156	0.2492
	L_eigen_centrality	0.176	0.173	0.201	0.201	18.693	< 0.0001	-1.945	0.0533	1.168	0.2444
	G_eigen_centrality	0.054	0.050	0.047	0.049	-3.756	0.0002	-2.566	0.0111	2.009	0.0461
	L_between_centrality	13.908	15.798	10.642	8.598	-3.490	0.0006	2.165	0.0317	-2.971	0.0034
	G_between_centrality	159.092	162.723	151.090	149.374	-1.091	0.2765	0.135	0.8927	-0.516	0.6067
	assortativity	0.145	0.195	0.174	0.324	0.726	0.4689	0.847	0.3984	1.829	0.0690
	participation_coef	0.830	0.814	0.829	0.832	-0.071	0.9432	-3.056	0.0026	2.427	0.0162

Supplementary Table 7

Statistical comparison of graph metrics in different thresholds during the finger tapping task

		Test					Retest			
		Left Somatosensory-motor	Right Somatosensory-motor	t-value	p-value		Left Somatosensory-motor	Right Somatosensory-motor	t-value	p-value
20 % threshold										
	L_density	0.447	0.349	-2.744	0.023		0.4283	0.315	-3.0870	0.013
	G_density	0.336	0.316	-1.167	0.273		0.2898	0.271	-1.1110	0.295
	L_global_efficiency	0.688	0.619	-2.597	0.029		0.6674	0.597	-2.3670	0.042
	L_nodal_efficiency	0.847	0.819	-1.229	0.250		0.8321	0.793	-2.5400	0.032
	G_nodal_efficiency	0.818	0.811	-0.377	0.715		0.8149	0.782	-3.2430	0.010
	L_shortest_path	1.738	1.924	1.834	0.100		1.7219	2.003	2.8700	0.019
	G_shortest_path	1.852	1.910	1.417	0.190		1.9330	2.005	1.8650	0.095
	L_clustering_coef	0.746	0.697	-1.714	0.121		0.7196	0.661	-2.7870	0.021
	G_clustering_coef	0.678	0.671	-0.320	0.756		0.6634	0.629	-2.7070	0.024
	L_eigen_centrality	0.165	0.125	-11.445	<0.0001		0.1632	0.124	-12.7090	<0.0001
	G_eigen_centrality	0.103	0.095	-0.936	0.374		0.1026	0.094	-0.8610	0.411
	L_between_centrality	20.469	40.352	5.377	0.000		19.1034	43.787	6.4520	0.000
	G_between_centrality	67.127	63.351	-0.993	0.347		64.1793	75.161	1.1690	0.273
	assortativity	0.094	0.243	2.465	0.036		0.1462	0.221	0.9530	0.366
	participation_coef	0.442	0.3709	-6.018	0.0002		0.4086	0.320	-13.4380	<0.0001
30 % threshold										
	L_density	0.531	0.429	-2.844	0.019		0.5315	0.401	-3.279	0.010
	G_density	0.424	0.400	-1.406	0.193		0.3903	0.362	-1.6360	0.136
	L_global_efficiency	0.752	0.685	-2.822	0.020		0.7393	0.666	-2.8440	0.019
	L_nodal_efficiency	0.868	0.847	-1.329	0.217		0.8667	0.831	-1.8900	0.091
	G_nodal_efficiency	0.853	0.841	-0.973	0.356		0.8411	0.821	-2.0320	0.073
	L_shortest_path	1.557	1.734	2.735	0.023		1.5372	1.779	3.1640	0.012
	G_shortest_path	1.656	1.715	1.981	0.079		1.7165	1.786	2.2610	0.050
	L_clustering_coef	0.776	0.730	-2.048	0.071		0.7701	0.704	-2.7150	0.024
	G_clustering_coef	0.720	0.709	-0.708	0.497		0.7024	0.676	-2.2130	0.054
	L_eigen_centrality	0.171	0.130	-19.792	<0.0001		0.1706	0.129	-18.0400	<0.0001
	G_eigen_centrality	0.107	0.100	-1.110	0.296		0.1089	0.098	-1.5940	0.145
	L_between_centrality	15.586	32.665	7.350	<0.0001		14.6345	34.591	6.8190	0.000
	G_between_centrality	52.762	49.685	-0.946	0.369		49.6813	59.531	1.9190	0.087
	assortativity	0.068	0.230	3.356	0.008		0.0680	0.205	2.2210	0.054
	participation_coef	0.4597	0.4034	-6.925	0.0001		0.4435	0.369	-11.6570	<0.0001
40 % threshold										
	L_density	0.606	0.499	-2.954	0.016		0.6165	0.482	-3.199	0.011
	G_density	0.503	0.474	-1.766	0.111		0.4811	0.447	-1.8420	0.099
	L_global_efficiency	0.797	0.734	-2.967	0.016		0.7984	0.721	-3.1300	0.012
	L_nodal_efficiency	0.901	0.865	-3.060	0.014		0.8848	0.861	-1.6320	0.137
	G_nodal_efficiency	0.875	0.861	-1.539	0.158		0.8674	0.851	-2.8120	0.020
	L_shortest_path	1.430	1.598	2.961	0.016		1.4178	1.616	3.3720	0.008
	G_shortest_path	1.532	1.589	2.296	0.047		1.5658	1.625	2.3330	0.045
	L_clustering_coef	0.809	0.755	-3.183	0.011		0.7985	0.748	-2.4310	0.038
	G_clustering_coef	0.752	0.740	-1.018	0.335		0.7432	0.722	-2.9100	0.017
	L_eigen_centrality	0.175	0.133	-25.198	<0.0001		0.1749	0.133	-20.9480	<0.0001
	G_eigen_centrality	0.110	0.103	-1.384	0.200		0.1119	0.101	-1.9700	0.080
	L_between_centrality	12.048	26.926	6.696	0.000		11.6345	27.504	6.8490	0.000
	G_between_centrality	43.265	41.129	-0.631	0.544		38.8286	47.778	2.7090	0.024
	assortativity	0.036	0.213	4.326	0.002		0.0380	0.183	2.3990	0.040
	participation_coef	0.4686	0.4257	-5.647	0.0003		0.4636	0.3953	-9.626	<0.0001

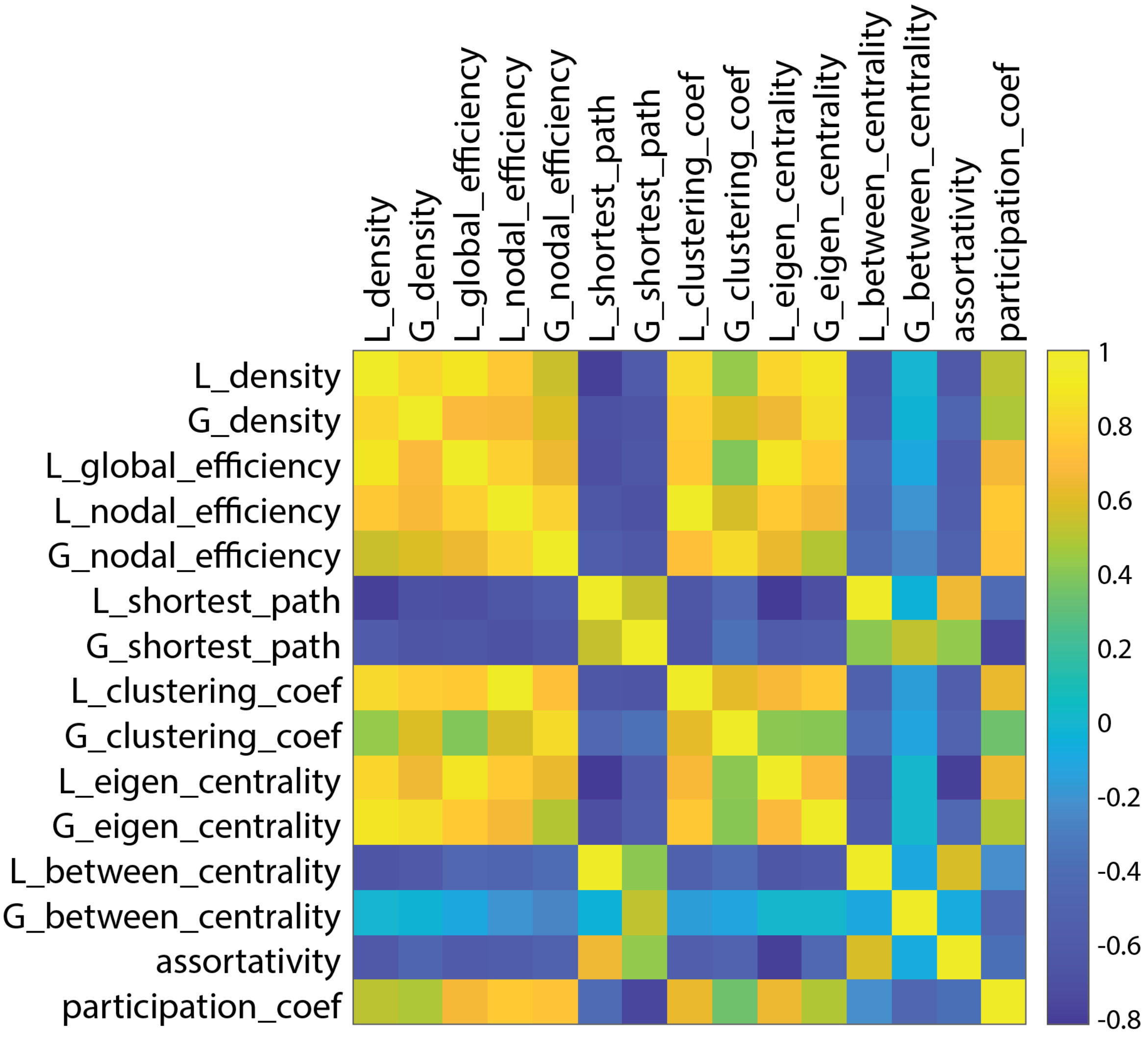

Supplementary Figure 1

Correlation of graph metrics

Bivariate correlations between graph metrics across participants from the word recognition module during the word comprehension task. L and G represent local and global graph metrics respectively.

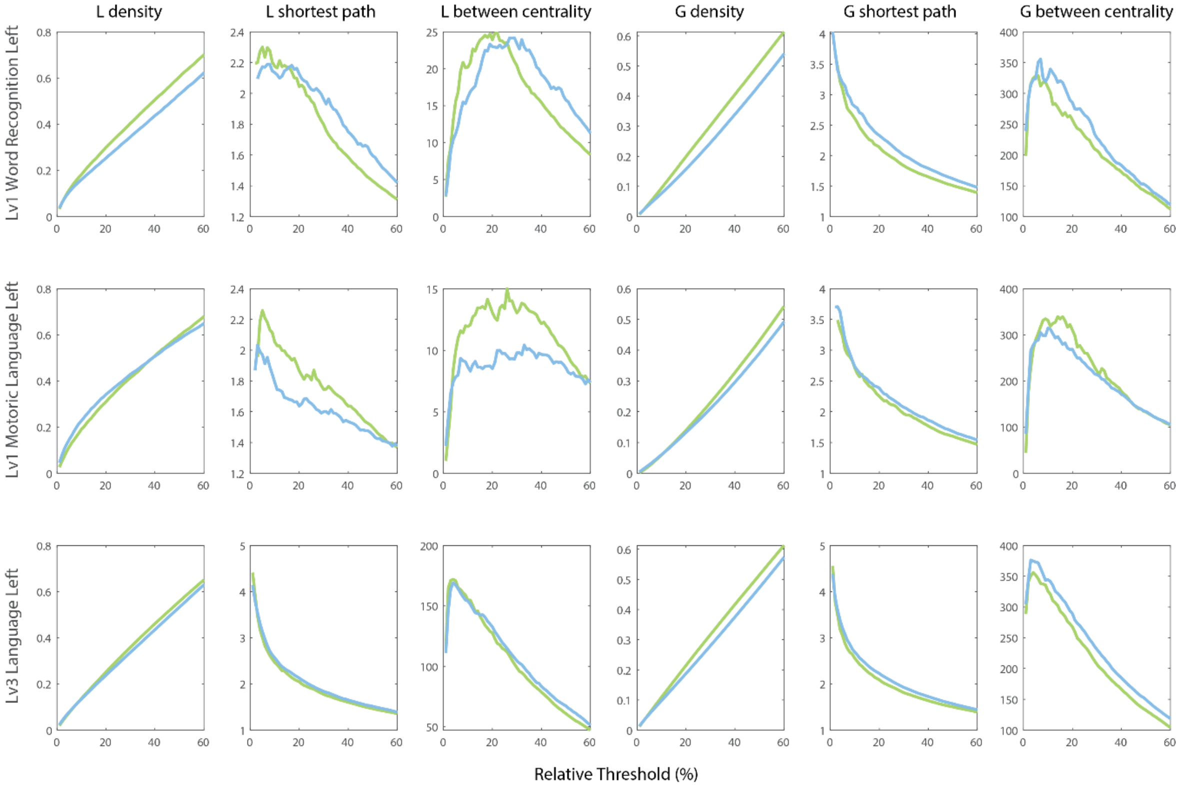

Supplemantary Figure 2

Plot of graph metrics for the entire range of relative thresholds from top 1% until 60% of correlations. Green and blue lines indicate the word comprehension and word generation tasks, respectively.
